# Calcium Orthophosphate-Containing Biocomposites and Hybrid Biomaterials for Biomedical Applications

**DOI:** 10.3390/jfb6030708

**Published:** 2015-08-07

**Authors:** Sergey V. Dorozhkin

**Affiliations:** Kudrinskaja sq. 1-155, Moscow 123242, Russia; E-Mail: sedorozhkin@yandex.ru

**Keywords:** calcium orthophosphates, hydroxyapatite, biocomposites, hybrid biomaterials, bone grafts, biomedical applications, tissue engineering

## Abstract

The state-of-the-art on calcium orthophosphate (CaPO_4_)-containing biocomposites and hybrid biomaterials suitable for biomedical applications is presented. Since these types of biomaterials offer many significant and exciting possibilities for hard tissue regeneration, this subject belongs to a rapidly expanding area of biomedical research. Through the successful combinations of the desired properties of matrix materials with those of fillers (in such systems, CaPO_4_ might play either role), innovative bone graft biomaterials can be designed. Various types of CaPO_4_-based biocomposites and hybrid biomaterials those are either already in use or being investigated for biomedical applications are extensively discussed. Many different formulations in terms of the material constituents, fabrication technologies, structural and bioactive properties, as well as both *in vitro* and *in vivo* characteristics have been already proposed. Among the others, the nano-structurally controlled biocomposites, those containing nanodimensional compounds, biomimetically fabricated formulations with collagen, chitin and/or gelatin, as well as various functionally graded structures seem to be the most promising candidates for clinical applications. The specific advantages of using CaPO_4_-based biocomposites and hybrid biomaterials in the selected applications are highlighted. As the way from a laboratory to a hospital is a long one and the prospective biomedical candidates have to meet many different necessities, the critical issues and scientific challenges that require further research and development are also examined.

## 1. Introduction

The fracture of bones due to various traumas or natural aging is a typical type of a tissue failure. An operative treatment frequently requires implantation of a temporary or a permanent prosthesis, which still is a challenge for orthopedic surgeons, especially in the cases of large bone defects. A fast aging of the population and serious drawbacks of natural bone grafts make the situation even worse; therefore, there is a high clinical demand for bone substitutes. Unfortunately, a medical application of xenografts (e.g., bovine bone) is generally associated with potential viral infections. In addition, xenografts have a low osteogenicity, an increased immunogenicity and, usually, resorb more rapidly than autogenous bone. Similar limitations are also valid for human allografts (*i.e.*, tissue transplantation between individuals of the same species but of non-identical genetic composition), where the concerns about potential risks of transmitting tumor cells, a variety of bacterial and viral infections, as well as immunological and blood group incompatibility are even stronger. Moreover, harvesting and conservation of allografts (exogenous bones) are additional limiting factors [1,2,3]. Autografts (endogenous bones) are still the “golden standard” among any substitution materials because they are osteogenic, osteoinductive, osteoconductive, completely biocompatible, non-toxic and do not cause any immunological problems (non-allergic). They contain viable osteogenic cells, bone matrix proteins and support bone growth. Usually, autografts are well accepted by the body and rapidly integrated into the surrounding bone tissues. Due to these reasons, they are used routinely for a long period with good clinical results [2,3,4,5]; however, it is fair to say on complication cases, those frequently happened in the past [6]. Unfortunately, a limited number of donor sites restrict the quantity of autografts harvested from the iliac crest or other locations of the patient’s own body. In addition, their medical application is always associated with additional traumas and scars resulting from the extraction of a donor tissue during a superfluous surgical operation, which requires further healing at the donation site and can involve long-term postoperative pain. Thus, any types of biologically derived transplants appear to be imperfect solutions, mainly due to a restricted quantity of donor tissues, donor site morbidity, as well as potential risks of an immunological incompatibility and disease transfer [7,8,9]. In this light, manmade materials (alloplastic or synthetic bone grafts) stand out as a reasonable option because they are easily available, might be processed and modified to suit the specific needs of a given application. What is more, there are no concerns about potential infections, immunological incompatibility, sterility and donor site morbidity. Therefore, investigations on artificial materials for bone tissue repair appear to be one of the key subjects in the field of biomaterials research for clinical applications [10,11].

Currently, there are several classes of synthetic bone grafting biomaterials for *in vivo* applications. The examples include natural coral, coral-derived materials, bovine porous demineralized bone, human demineralized bone matrix, bioactive glasses, glass-ceramics and CaPO_4_ [12,13]. Among them, porous bioceramics made of CaPO_4_ appear to be very prominent due to both the excellent biocompatibility and bonding ability to living bone in the body. This is directly related to the fact that the inorganic material of mammalian calcified tissues, *i.e.*, of bone and teeth, consists of CaPO_4_ [14,15]. Due to this reason, other artificial materials are normally encapsulated by fibrous tissue, when implanted in body defects, while CaPO_4_ are not. Many types of CaPO_4_-based bioceramics with different chemical composition are already on the market [16,17]. Unfortunately, as for any ceramic material, CaPO_4_ bioceramics alone lack the mechanical and elastic properties of the calcified tissues. Namely, scaffolds made of CaPO_4_ only suffer from a low elasticity, a high brittleness, a poor tensile strength, a low mechanical reliability and fracture toughness, which leads to various concerns about their mechanical performance after implantation. Besides, in many cases, it is difficult to form CaPO_4_ bioceramics into the desired shapes [16,17].

The superior strength and partial elasticity of biological calcified tissues (e.g., bones) are due to the presence of bioorganic polymers (mainly, collagen type I fibers) rather than to a natural ceramic (mainly, a poorly crystalline ion-substituted calcium-deficient hydroxyapatite (CDHA), often referred to as “biological apatite”) phase [18,19,20,21]. The elastic collagen fibers are aligned in bone along the main stress directions. The biochemical composition of bones is given in Table 1 [22]. A decalcified bone becomes very flexible being easily twisted, whereas a bone without collagen is very brittle; thus, the inorganic nano-sized crystals of biological apatite provide with the hardness and stiffness, while the bioorganic fibers are responsible for the elasticity and toughness. In bones, both types of materials integrate each other into a nanometric scale in such a way that the crystallite size, fibers orientation, short-range order between the components, *etc.*, determine its nanostructure and therefore the function and mechanical properties of the entire composite. From the mechanical point of view, bone is a tough material at low strain rates but fractures more like a brittle material at high strain rates; generally, it is rather weak in tension and shear, particularly along the longitudinal plane. Besides, bone is an anisotropic material because its properties are directionally dependent [18,19,20,21].

**Table 1:** The biochemical composition of bones [22]. The composition is varied from species to species and from bone to bone.

Inorganic Phases	wt %	Bioorganic Phases	wt %
CaPO_4_ (biological apatite)	~60	collagen type I	~20
water	~9	non-collagenous proteins: osteocalcin, osteonectin, osteopontin, thrombospondin, morphogenetic proteins, sialoprotein, serum proteins	~3
carbonates	~4	other traces: polysaccharides, lipids, cytokines	balance
citrates	~0.9	primary bone cells: osteoblasts, osteocytes, osteoclasts	balance
sodium	~0.7		
magnesium	~0.5		
other traces: Cl^−^, F^−^, K^+^ Sr^2+^, Pb^2+^, Zn^2+^, Cu^2+^, Fe^2+^	balance		

It remains a great challenge to design the ideal bone graft that emulates the nature’s own structures or functions. Certainly, the successful design requires an appreciation of the bones’ structure. According to expectations, the ideal bone graft should be benign, available in a variety of forms and sizes, all with sufficient mechanical properties for use in load-bearing sites, form a chemical bond at the bone/implant interface, as well as be osteogenic, osteoinductive, osteoconductive, biocompatible, completely biodegradable at the expense of bone growth and moldable to fill and restore bone defects [23,24]. Further, it should resemble the chemical composition of bones (thus, the presence of CaPO_4_ is mandatory), exhibit contiguous porosity to encourage invasion by the live host tissue, as well as possess both viscoelastic and semi-brittle behavior, as bones do [25,26,27]. Moreover, the degradation kinetics of the ideal implant should be adjusted to the healing rate of the human tissue with absence of any chemical or biological irritation and/or toxicity caused by substances, which are released due to corrosion or degradation. Ideally, the combined mechanical strength of the implant and the ingrowing bone should remain constant throughout the regenerative process. Furthermore, the substitution implant material should not disturb significantly the stress environment of the surrounding living tissue [28]. Finally, there is an opinion, that in the case of a serious trauma, bone should fracture rather than the implant [23]. A good sterilizability, storability and processability, as well as a relatively low cost are also of a great importance to permit a clinical application. Unfortunately, no artificial biomaterial is yet available, which embodies all these requirements and unlikely it will appear in the nearest future. To date, most of the available biomaterials appear to be either predominantly osteogenic or osteoinductive or else purely osteoconductive [1].

Careful consideration of the bone type and mechanical properties are needed to design bone substitutes. Indeed, in high load-bearing bones such as the femur, the stiffness of the implant needs to be adequate, not too stiff to result in strain shielding, but rigid enough to present stability. However, in relatively low load-bearing applications such as cranial bone repairs, it is more important to have stability and the correct three-dimensional shapes for aesthetic reasons. One of the most promising alternatives is to apply materials with similar composition and nanostructure to that of bone tissue. Mimicking the structure of calcified tissues and addressing the limitations of the individual materials, development of organic-inorganic hybrid biomaterials provides excellent possibilities for improving the conventional bone implants. In this sense, suitable biocomposites of tailored physical, biological and mechanical properties with the predictable degradation behavior can be prepared combining biologically relevant CaPO_4_ with bioresorbable polymers [29]. As a rule, the general behavior of such biocomposites is dependent on nature, structure and relative contents of the constitutive components, although other parameters such as the preparation conditions also determine the properties of the final materials. Currently, CaPO_4_ is incorporated as either a filler or a coating (or both) either into or onto a biodegradable polymer matrix, in the form of particles or fibers, and are increasingly considered for using as bone tissue engineering scaffolds due to their improved physical, biologic and mechanical properties [30,31,32,33]. In addition, such biocomposites could fulfill general requirements to the next generation of biomaterials, those should combine the bioactive and bioresorbable properties to activate *in vivo* mechanisms of tissue regeneration, stimulating the body to heal itself and leading to replacement of the implants by the regenerating tissue. Thus, through the successful combinations of ductile polymer matrixes with hard and bioactive particulate bioceramic fillers, optimal materials can be designed and, ideally, this approach could lead to a superior construction to be used as either implants or posterior dental restorative material [29,34].

A lint-reinforced plaster was the first composite used in clinical orthopedics as an external immobilizer (bandage) in the treatment of bone fracture by Mathijsen in 1852 [35], followed by Dreesman in 1892 [36]. A great progress in the clinical application of various types of composite materials has been achieved since then. Based on both the past experience and the newly gained knowledge, various composite materials with tailored mechanical and biological performance can be manufactured and used to meet various clinical requirements [37]. However, this review presents only a brief history and advances in the field of CaPO_4_-based biocomposites and hybrid biomaterials suitable for biomedical application. The majority of the reviewed literature is restricted to the recent publications; a limited number of papers published in the XX-th century have been cited. Various aspects of the material constituents, fabrication technologies, structural and bioactive properties, as well as phase interaction have been considered and discussed in details. Finally, several critical issues and scientific challenges that are needed for further advancement are outlined.

## 2. General Information and Knowledge

According to Wikipedia, the free encyclopedia, “*composite materials* (or *composites* for short) are engineered materials made from two or more constituent materials with significantly different physical or chemical properties and which remain separate and distinct on a macroscopic level within the finished structure” [38]. Thus, composites are always heterogeneous. Furthermore, the phases of any composite retain their identities and properties, and are bonded, which is why an interface is maintained between them. This provides improved specific or synergistic characteristics that are not obtainable by any of the original phases alone [39]. Following the point of view of some predecessors, we also consider that “for the purpose of this review, composites are defined as those having a distinct phase distributed through their bulk, as opposed to modular or coated components” [40] (p. 1329). For this reason, with a few important exceptions, the structures obtained by soaking of various materials in supersaturated solutions containing ions of calcium and orthophosphate (e.g., Refs. [41,42,43,44]), those obtained by coating of various materials by CaPO_4_ (reviewed in Refs. [45,46,47]), as well as CaPO_4_ coated by other compounds [48,49,50,51] have not been considered; however, composite coatings have been considered. Occasionally, porous CaPO_4_ scaffolds filled by cells inside the pores [52,53,54,55], as well as CaPO_4_ impregnated by biologically active substances [56,57] are also defined as composites and/or hybrids; nevertheless, such structures have not been considered either.

In any composite, there are two major categories of constituent materials: a matrix (or a continuous phase) and (a) dispersed phase(s). To create a composite, at least one portion of each type is required. General information on the major fabrication and processing techniques might be found elsewhere [40,58]. The continuous phase is responsible for filling the volume, as well as it surrounds and supports the dispersed material(s) by maintaining their relative positions. The dispersed phase(s) is (are) usually responsible for enhancing one or more properties of the matrix. Most of the composites target an enhancement of mechanical properties of the matrix, such as stiffness and strength; however, other properties, such as erosion stability, transport properties (electrical or thermal), radiopacity, density or biocompatibility might also be of a great interest. This synergism produces the properties, which are unavailable from the individual constituent materials [58,59]. What’s more, by controlling the volume fractions and local and global arrangement of the dispersed phase, the properties and design of composites can be varied and tailored to suit the necessary conditions. For example, in the case of ceramics, the dispersed phase serves to impede crack growth. In this case, it acts as reinforcement. A number of methods, including deflecting crack tips, forming bridges across crack faces, absorbing energy during pullout and causing a redistribution of stresses in regions, adjacent to crack tips, can be used to accomplish this [60]. Other factors to be considered in composites are the volume fraction of the dispersed phase(s), its (their) orientation and homogeneity of the overall composite. For example, higher volume fractions of reinforcement phases tend to improve the mechanical properties of the composites, while continuous and aligned fibers best prevent crack propagation with the added property of anisotropic behavior. From a structural point of view, composites are anisotropic in nature: their mechanical properties are different in different directions. Furthermore, the uniform distribution of the dispersed phase is also desirable, as it imparts consistent properties to the composite [38,58,59].

In general, composites might be simple, complex, graded and hierarchical. The term “a simple composite” is referred to the composites those result from the homogeneous dispersion of one dispersed phase throughout a matrix. The term “a complex composite” is referred to the composites those result from the homogeneous dispersion of several dispersed phases throughout one matrix. The term “a graded composite” is referred to the composites those result from the intentionally structurally inhomogeneous dispersion of one or several dispersed phases throughout one matrix. The term “a hierarchical composite” is referred to the cases, when fine entities of either a simple or a complex composite is somehow aggregated to form coarser ones (e.g., granules or particles) which afterwards are dispersed inside another matrix to produce the second hierarchical scale of the composite structure. There is another set of four types of composites: (i) fibrous composites, where the fibers are in a matrix; (ii) laminar composites, in which the phases are in layers; (iii) particulate composites, where the particles or flakes are in a matrix; and (iv) hybrid composites, which are combinations of any of the above. Still other classification type of the available composites is based on the matrix materials (metals, ceramics and polymers) [37].

In most cases, three interdependent factors must be considered in designing of any composite: (i) a selection of the suitable matrix and dispersed materials, (ii) a choice of appropriate fabrication and processing methods, (iii) both internal and external design of the device itself [40]. Furthermore, any composite must be formed to shape. To do this, the matrix material can be added before or after the dispersed material has been placed into a mold cavity or onto the mold surface. The matrix material experiences a melding event, that, depending upon the nature of the matrix material, can occur in various ways such as chemical polymerization, setting, curing or solidification from a melted state. Due to a general inhomogeneity, the physical properties of many composite materials are not isotropic but rather orthotropic (*i.e.*, there are different properties or strengths in different orthogonal directions) [38,58,59].

In order to prepare any type of a composite, at least, two different materials must be mixed. Thus, a phase miscibility phenomenon appears to be of the paramount importance [61,62]. Furthermore, the interfacial strength among the phases is a very important factor because a lack of adhesion among the phases will result in an early failure at the interface and thus in a decrease in the mechanical properties, especially the tensile strength. From a chemical point of view, we can distinguish several types of the interaction among the composite components: materials with strong (covalent, coordination, ionic) interactions; those with weak interactions (van der Waals forces, hydrogen bonds, hydrophilic-hydrophobic balance) or without chemical interactions among the components [63]. Wetting is also important in bonding or adherence of the materials. It depends on the hydrophilicity or polarity of the filler(s) and the available polar groups of the matrix.

Regarding biocomposites, they are defined as nontoxic composites able to interact well with the human body *in vivo* and, ideally, contain one or more component(s) that stimulate(s) the healing process and uptake of the implant [64]. Thus, for biocomposites the biological compatibility appears to be more important than any other type of compatibility [37,65,66,67]. Interestingly that according to the databases, the first paper with the term “biocomposite” in the title was published in 1987 [68] and the one containing a combination of terms “biocomposite” and HA in the title was published in 1991 [69]. Thus, the subject of CaPO_4_-based biocomposites and hybrid biomaterials appears to be quite new. The most common properties from the bioorganic and inorganic domains to be combined in biocomposites have been summarized in Table 2 [24]. For general advantages of the modern CaPO_4_-based biocomposites over CaPO_4_ bioceramics and bioresorbable polymers individually, the interested readers are advised to get through “Composite materials strategy” section of Ref. [29].

**Table 2:** General respective properties from the bioorganic and inorganic domains, to be combined in various composites and hybrid materials [24].

Inorganic	Bioorganic
hardness, brittleness	elasticity, plasticity
high density	low density
thermal stability	permeability
hydrophilicity	hydrophobicity
high refractive index	selective complexation
mixed valence slate (red-ox)	chemical reactivity
strength	bioactivity

## 3. The Major Constituents

### 3.1. CaPO_4_

CaPO_4_ were first mentioned in 1769 as the major constituents of bones and have been investigated since then [70,71]. The main driving force behind the use of CaPO_4_ as bone substitute materials is their chemical similarity to the mineral component of mammalian bones and teeth [14,15,16]. As a result, in addition to being non-toxic, they are biocompatible, not recognized as foreign materials in the body and, most importantly, both exhibit bioactive behavior and integrate into living tissue by the same processes active in remodeling healthy bone. This leads to an intimate physicochemical bond between the implants and bone, termed osteointegration. More to the point, CaPO_4_ are also known to support osteoblast adhesion and proliferation. Even so, the major limitations to use CaPO_4_ as load-bearing biomaterials are their mechanical properties; namely, they are brittle with poor fatigue resistance [23]. The poor mechanical behavior is even more evident for highly porous ceramics and scaffolds because porosity greater than 100 µm is considered as the requirement for proper vascularization and bone cell colonization [72,73]. That is why, in biomedical applications CaPO_4_ are used primarily as fillers and coatings [16].

The complete list of known CaPO_4_, including their standard abbreviations and the major properties, is given in Table 3, while the detailed information on CaPO_4_, might be found in special books and monographs [16,74,75,76,77,78].

**Table 3:** Existing CaPO_4_ and their major properties [16].

Ca/P Molar Ratio	Compound	Formula	Solubility at 25 °C, −log (*K*_s_)	Solubility at 25 °C, g/L	pH Stability Range in Aqueous Solutions at 25 °C
0.5	Monocalcium phosphate monohydrate (MCPM)	Ca(H_2_PO_4_)_2_·H_2_O	1.14	~18	0.0–2.0
0.5	Monocalcium phosphate anhydrous (MCPA or MCP)	Ca(H_2_PO_4_)_2_	1.14	~17	^[c]^
1.0	Dicalcium phosphate dihydrate (DCPD), mineral brushite	CaHPO_4_·2H_2_O	6.59	~0.088	2.0–6.0
1.0	Dicalcium phosphate anhydrous (DCPA or DCP), mineral monetite	CaHPO_4_	6.90	~0.048	^[c]^
1.33	Octacalcium phosphate (OCP)	Ca_8_(HPO_4_)_2_(PO_4_)_4_·5H_2_O	96.6	~0.0081	5.5–7.0
1.5	α-Tricalcium phosphate (α-TCP)	α-Ca_3_(PO_4_)_2_	25.5	~0.0025	^[a]^
1.5	β-Tricalcium phosphate (β-TCP)	β-Ca_3_(PO_4_)_2_	28.9	~0.0005	^[a]^
1.2–2.2	Amorphous calcium phosphates (ACP)	Ca*_x_*H*_y_*(PO_4_)*_z_*·*n*H_2_O, *n* = 3–4.5; 15%–20% H_2_O	^[b]^	^[b]^	~5–12 ^[d]^
1.5–1.67	Calcium-deficient hydroxyapatite (CDHA or Ca-def HA) ^[e]^	Ca_10−*x*_(HPO_4_)*_x_*(PO_4_)_6−*x*_(OH)_2−*x*_ (0 < *x* < 1)	~85	~0.0094	6.5–9.5
1.67	Hydroxyapatite (HA, HAp or OHAp)	Ca_10_(PO_4_)_6_(OH)_2_	116.8	~0.0003	9.5–12
1.67	Fluorapatite (FA or FAp)	Ca_10_(PO_4_)_6_F_2_	120.0	~0.0002	7–12
1.67	Oxyapatite (OA, OAp or OXA) ^[f]^, mineral voelckerite	Ca_10_(PO_4_)_6_O	~69	~0.087	^[a]^
2.0	Tetracalcium phosphate (TTCP or TetCP), mineral hilgenstockite	Ca_4_(PO_4_)_2_O	38–44	~0.0007	^[a]^

^[a]^ These compounds cannot be precipitated from aqueous solutions. ^[b]^ Cannot be measured precisely. However, the following values were found: 25.7 ± 0.1 (pH = 7.40), 29.9 ± 0.1 (pH = 6.00), 32.7 ± 0.1 (pH = 5.28). The comparative extent of dissolution in acidic buffer is: ACP >> α-TCP >> β-TCP > CDHA >> HA > FA. ^[c]^ Stable at temperatures above 100 °C. ^[d]^ Always metastable. ^[e]^ Occasionally, it is called “precipitated HA (PHA)”. ^[f]^ Existence of OA remains questionable.

### 3.2. Polymers

Polymers are a class of materials consisting of large molecules, often containing many thousands of small units, or monomers, joined together chemically to form one giant chain. In this respect, polymers are comparable with major functional components of the biological environment: lipids, proteins and polysaccharides. They differ from each other in chemical composition, molecular weight, polydispersity, crystallinity, hydrophobicity, solubility and thermal transitions. Besides, their properties can be fine-tuned over a wide range by varying the type of polymer, chain length, as well as by copolymerization or blending of two or more polymers [79,80]. Opposite to ceramics, polymers exhibit substantial viscoelastic properties and easily can be fabricated into complex structures, such as sponge-like sheets, gels or complex structures with intricate porous networks and channels [81]. Being X-ray transparent and non-magnetic polymeric materials are fully compatible with the modern diagnostic methods such as computed tomography and magnetic resonance imaging. Unfortunately, most of them are unable to meet the strict demands of the *in vivo* physiological environment. Namely, the main requirements to polymers suitable for biomedical applications are that they must be biocompatible, not eliciting an excessive or chronic inflammatory response upon implantation and, for those that degrade, that they breakdown into non-toxic products only. Unfortunately, polymers, for the most part, lack rigidity, ductility and ultimate mechanical properties required in load bearing applications. Thus, despite their good biocompatibility, many of the polymeric materials are mainly used for soft tissue replacements (such as skin, blood vessel, cartilage, ligament replacement, *etc*). Moreover, the sterilization processes (autoclave, ethylene oxide and ^6^°Co irradiation) may affect the polymer properties [82].

There is a variety of biocompatible polymers suitable for biomedical applications [83,84,85]. For example, polyacrylates, poly(acrylonitrile*-co-*vinylchloride) and polylysine have been investigated for cell encapsulation and immunoisolation [86,87]. Polyorthoesters and PCL have been investigated as drug delivery devices, the latter for long-term sustained release because of their slow degradation rates [88]. PCL is a hydrolytic polyester having appropriate resorption period and releases nontoxic byproducts upon degradation [89,90]. PU is in use in engineering of both hard and soft tissues, as well as in nanomedicine [91]. Polymers considered for orthopedic purposes include polyanhydrides, which have also been investigated as delivery devices (due to their rapid and well-defined surface erosion), for bone augmentation or replacement since they can be photopolymerized *in situ* [88,92,93]. To overcome their poor mechanical properties, they have been co-polymerized with imides or formulated to be crosslinkable *in situ* [93]. Other polymers, such as polyphosphazenes, can have their properties (e.g., degradation rate) easily modified by varying the nature of their side groups and have been shown to support osteoblast adhesion, which makes them candidate materials for skeletal tissue regeneration [93]. PPF has emerged as a good bone replacement material, exhibiting good mechanical properties (comparable to trabecular bone), possessing the capability to crosslink *in vivo* through the C=C bond and being hydrolytically degradable. It has also been examined as a material for drug delivery devices [88,92,93,94,95]. Polycarbonates have been suggested as suitable materials to make scaffolds for bone replacement and have been modified with tyrosine-derived amino acids to render them biodegradable [88,96]. Polydioxanone has been also tested for biomedical applications [97]. PMMA is widely used in orthopedics, as a bone cement for implant fixation, as well as to repair certain fractures and bone defects, for example, osteoporotic vertebral bodies [98,99]. However, PMMA sets by a polymerization of toxic monomers, which also evolves significant amounts of heat that damages tissues. Moreover, it is neither degradable nor bioactive, does not bond chemically to bones and might generate particulate debris leading to an inflammatory foreign body response [92,100]. A number of other non-degradable polymers applied in orthopedic surgery include PE in its different modifications such as low density PE, HDPE and ultrahigh molecular weight PE (used as the articular surface of total hip replacement implants [101,102]), polyethylene terepthalate and PP, which are applied to repair knee ligaments [103]. Polyactive™, a block copolymer of PEG and PBT, was also considered for biomedical application [104,105,106]. Cellulose [107,108] and its esters [109,110] are also popular. Finally yet importantly, polyethylene oxide, PHB and blends thereof have also been tested for biomedical applications [29].

Nonetheless, the most popular synthetic polymers used in medicine are the linear aliphatic poly(α-hydroxyesters) such as PLA, PGA and their copolymers—PLGA (Table 4). These materials have been extensively studied; they appear to be the only synthetic and biodegradable polymers with an extensive FDA approval history [29,93,111,112]. They are biocompatible, mostly non-inflammatory, as well as degrade *in vivo* through hydrolysis and possible enzymatic action into products that are removed from the body by regular metabolic pathways [88,93,113]. Besides, they might be used for drug delivery purposes [114]. Poly(α-hydroxyesters) have been investigated as scaffolds for replacement and regeneration of a variety of tissues, cell carriers, controlled delivery devices for drugs or proteins (e.g., growth factors), membranes or films, screws, pins and plates for orthopedic applications [88,93,115,116]. Additionally, the degradation rate of PLGA can be adjusted by varying the amounts of the two component monomers (Table 4), which in orthopedic applications can be exploited to create materials that degrade in concert with bone ingrowth [117]. Furthermore, PLGA is known to support osteoblast migration and proliferation [93,118], which is a necessity for bone tissue regeneration. Unfortunately, such polymers on their own, though they reduce the effect of stress-shielding, are too weak to be used in load bearing situations and are only recommended in certain clinical indications, such as ankle and elbow fractures [113]. In addition, they exhibit bulk degradation, leading to both a loss in mechanical properties and lowering of the local solution pH that accelerates further degradation in an autocatalytic manner. As the body is unable to cope with the vast amounts of implant degradation products, this might lead to an inflammatory foreign body response. Finally, poly(α-hydroxyesters) do not possess the bioactive and osteoconductive properties [93,119].

Several classifications of the biomedically relevant polymers are possible. For example, some authors distinguish between synthetic polymers like PE, PMMA, PLA, PGA, PCL, *etc.*, and polymers of biological origin, which comprise polysaccharides (starch, alginate, chitin/chitosan [120,121], gellan gum, cellulose, hyaluronic acid and its derivatives), proteins (soy, collagen, gelatin, fibrin, silk) and a variety of biofibers, such as lignocellulosic natural fibers [122,123]. Among them, natural polymers often posses highly organized structures and may contain an extracellular substance, called ligand, which is necessary to bind with cell receptors. However, they always contain various impurities, which should be removed prior use. As synthetic polymers can be produced under the controlled conditions, they in general exhibit predictable and reproducible mechanical and physical properties such as tensile strength, elastic modulus and degradation rate. Control of impurities is a further advantage of synthetic polymers. Other authors differentiate between resorbable or biodegradable (e.g., poly(α-hydroxyesters), polysaccharides and proteins) and non-resorbable (e.g., PE, PP, PMMA and cellulose) polymers [123]. Furthermore, polymeric materials can be broadly classified as thermoplastics and thermosets. For example, HDPE and PEEK are the examples of thermoplastics, while polydimethylsiloxane and PMMA are the examples of thermosets [82]. The list of synthetic biodegradable polymers used for biomedical application as scaffold materials is available as Table 1 in Ref. [123], while further details on polymers suitable for biomedical applications are available in literature [82,116,124,125,126,127,128,129] where the interested readers are referred to. Good reviews on the synthesis of different biodegradable polymers [130], as well as on the experimental trends in polymer composites [131] are available elsewhere.

**Table 4:** Major properties of several FDA (Food and Drug Administration) approved biodegradable polymers [111]. *T*_g_, glass transition temperature; *T*_m_, melting point.

Polymer	Thermal Properties (°C)	Tensile Modulus (GРa)	Degradation Time (Months)
polyglycolic acid (PGA)	*T*_g_ = 35–40	7.06	6–12 (strength loss within 3 weeks)
	*T*_m_ = 225–230		
L-polylactic acid (LPLA)	*T*_g_ = 60–65	2.7	>24
	*T*_m_ = 173–178		
D,L-polylactic acid (DLPLA)	*T*_g_ = 55–60	1.9	12–16
	amorphous		
85/15 D,L-polylactic*-co-*glycolic acid (85/15 DLPLGA)	*T*_g_ = 50–55	2.0	5–6
	amorphous		
75/25 D,L-polylactic*-co-*glycolic acid (75/25 DLPLGA)	*T*_g_ = 50–55	2.0	4–5
	amorphous		
65/35 D,L-polylactic*-co-*glycolic acid (65/35 DLPLGA)	*T*_g_ = 45–50	2.0	3–4
	amorphous		
50/50 D,L-polylactic*-co-*glycolic acid (50/50 DLPLGA)	*T*_g_ = 45–50	2.0	1–2
	amorphous		
poly(ε-caprolactone) (PCL)	*T*_g_ = (−60)–(−65)	0.4	>24
	*T*_m_ = 58–63		

### 3.3. Inorganic Materials and Compounds

#### 3.3.1. Metals

Titanium (Ti) is one of the best biocompatible metals and used most widely as implant [132,133]. Besides, there are other metallic implants made of pure Zr, Hf, V, Nb, Ta, Re, Ni, Fe, Cu, Ag, stainless steels and various alloys suitable for biomedical application [134,135,136]. Recent studies revealed even a greater biomedical potential of porous metals [137,138,139]. The metallic implants provide the necessary strength and toughness that are required in load-bearing parts of the body and, due to these advantages, metals will continue to play an important role as orthopedic biomaterials in the future, even though there are concerns with regard to the release of certain ions from and corrosion products of metallic implants. Of course, neither metals nor alloys are biomimetic (the term biomimetic can be defined as a processing technique that either mimics or inspires the biological mechanism, in part or whole [140]) in terms of chemical composition because there are no elemental metals in the human body. In addition, even biocompatible metals are bioinert: while not rejected by the human body, any metallic implants cannot actively interact with the surrounding tissues. Nevertheless, in some cases (especially when they are coated by CaPO_4_ [47]) the metallic implants can show a reasonable biocompatibility [141]. Until recently, only permanent implants were made of metals and alloys, in which degradation or corrosion was not desirable. However, during recent years a number of magnesium implants have been proposed which are aimed to degrade in the body in order to make room for ingrowing bones [142,143].

#### 3.3.2. Glasses and Glass-Ceramics

Special types of both glasses [144,145] and glass-ceramics [146,147] are also suitable materials for biomedical applications and a special Na_2_O–CaO–SiO_2_–P_2_O_5_ formulation named Bioglass^®^ [148,149,150] is the most popular among them. They are produced via standard glass production techniques and require pure raw materials. Bioglass^®^ is a biocompatible and osteoconductive biomaterial. It bonds to bone without an intervening fibrous connective tissue interface and, due to these properties, it has been widely used for filling bone defects. The primary shortcoming of Bioglass^®^ is mechanical weakness and low fracture toughness due to an amorphous two-dimensional glass network. The bending strength of most Bioglass^®^ compositions is in the range of 40–60 MPa, which is not suitable for major load-bearing applications. Making porosity in Bioglass^®^-based scaffolds is beneficial for even better resorption and bioactivity [148,149,150].

By heat treatment, a suitable glass can be converted into glass-crystal composites containing crystalline phase(s) of controlled sizes and contents. The resultant glass-ceramics can have superior mechanical properties to the parent glass as well as to sintered crystalline ceramics [146,147]. The bioactive A-W glass-ceramics is made from the parent glass in the pseudoternary system 3CaO·P_2_O_5_–CaO·SiO_2_–MgO·CaO·2SiO_2_, which is produced by a conventional melt-quenching method. The bioactivity of A-W glass-ceramics is much higher than that of sintered HA. It possesses excellent mechanical properties and has therefore been used clinically for iliac and vertebrae prostheses and as intervertebral spacers [151,152].

#### 3.3.3. Ceramics

Metal oxide ceramics, such as alumina (Al_2_O_3_, high purity, polycrystalline, fine grained) zirconia (ZrO_2_) and some other oxides (e.g., TiO_2_, SiO_2_) have been widely studied due to their bioinertness, excellent tribological properties, high wear resistance, fracture toughness and strength, as well as a relatively low friction [148,153,154]. Among them, due to transformation from the tetragonal to the monoclinic phase, a volume change occurs when pure zirconia is cooled down, which causes cracking of the zirconia ceramics [155]. Therefore, additives such as calcia (CaO), magnesia (MgO) and yttria (Y_2_O_3_) must be mixed with zirconia to stabilize the material in either the tetragonal or the cubic phase. Such material is called PSZ [156,157]. However, the brittle nature of any ceramics has limited their scope of clinical applications and hence more research is needed to improve their properties.

#### 3.3.4. Carbon

Due to its bioinertness, excellent tribological properties, fracture toughness and strength, as well as a low friction, elemental carbon has been used as a biomaterial, at least, since 1972 [158]. Applications include orthopedic prostheses, vitreous carbon roots for replacement teeth, structural skeletal extensions, bone bridges and hip prostheses. Biomedical properties of amorphous carbon were studied as well [159]. However, current trends represent investigations on biomedical applications of nanodimentional carbon, such as nanotubes [160,161].

Carbon nanotubes with their small dimensions, a high aspect (length to diameter) ratio as well as the exceptional mechanical properties, including extreme flexibility and strength, significant resistance to bending, high resilience and the ability to reverse any buckling of the tube, have the excellent potential to accomplish necessary mechanical properties. The studies revealed that they might possess some bioactivity [162,163]. However, non-functionalized carbon nanotubes tend to agglomerate and form bundles. Besides, they are soluble in neither water nor organic solvents. Luckily, chemical functionalization allows carbon nanotubes to be dispersed more easily, which can improve interfacial bonding with other components of the composites [164]. Furthermore, functionalization of carbon nanotubes with carboxylic groups was found to confer a capacity to induce calcification similar to woven bones [165]. Interestingly that carbon nanotubes might be functionalized by *in situ* deposition of CDHA on their surface [166].

## 4. Biocomposites and Hybrid Biomaterials Containing CaPO_4_

Generally, the available CaPO_4_-containing biocomposites and hybrid biomaterials suitable for biomedical applications might be divided into several (partly overlapping) broad areas:

- biocomposites with polymers;
- self-setting formulations;
- formulations based on nanodimensional CaPO_4_ and nanodimensional biocomposites;
- biocomposites with collagen;
- formulations with other bioorganic compounds and/or biological macromolecules;
- injectable bone substitutes (IBS);
- biocomposites with inorganic compounds, carbon and metals;
- functionally graded formulations;
- biosensors.

The majority of them were developed following a bone-analogue concept in attempts to mimic natural bones. The details on each area are provided below.

### 4.1. Biocomposites with Polymers

Typically, the polymeric components of biocomposites and hybrid biomaterials comprise polymers that both have shown a good biocompatibility and are routinely used in surgical applications. In general, since polymers have a low modulus (2–7 GPa, as the maximum) as compared to that of bone (3–30 GPa), CaPO_4_ bioceramics need to be loaded at a high weight % ratio. Besides, general knowledge on composite mechanics suggests that any high aspect ratio particles, such as whiskers or fibers, significantly improve the modulus at a lower loading. Thus, some attempts have been already performed to prepare biocomposites containing whisker-like [167,168,169,170,171,172,173] or needle-like [174,175,176,177] CaPO_4_, as well as CaPO_4_ fibers [178].

The history of implantable CaPO_4_/polymer formulations started in 1981 (however, a more general topic “ceramic-plastic material as a bone substitute” is, at least, 18 years older [179]) from the pioneering study by Prof. William Bonfield and colleagues at Queen Mary College, University of London, performed on HA/PE blends [180,181]. That initial study introduced a bone-analogue concept, when proposed biocomposites comprised a polymer ductile matrix of PE and a ceramic stiff phase of HA, and was substantially extended and developed in further investigations by that research group [66,182,183,184,185,186,187,188,189,190,191,192]. More recent studies included investigations on the influence of surface topography of HA/PE composites on cell proliferation and attachment [193,194,195,196]. The material is composed of a particular combination of HA particles at a volume loading of ~40% uniformly dispensed in a HDPE matrix. The idea was to mimic bones by using a polymeric matrix that can develop a considerable anisotropic character through adequate orientation techniques reinforced with a bone-like bioceramics that assures both a mechanical reinforcement and a bioactive character of the composite. Following FDA approval in 1994, in 1995 this material has become commercially available under the trade-name HAPEX™ (Smith and Nephew Richards, Bartlett, TN, USA), and to date it has been implanted in over 300,000 patients with the successful results. It remains the only clinically successful bioactive composite, which was a major step in the implant field [148,197]. The major production stages of HAPEX™ include blending, compounding and centrifugal milling. A bulk material or device is then created from this powder by compression and injection molding [37]. Besides, HA/HDPE biocomposites might be prepared by a hot rolling technique that facilitated uniform dispersion and blending of the reinforcements in the matrix [198]. In addition, PP might be used instead of PE [199,200,201,202].

A mechanical interlock between the both phases of HAPEX™ is formed by shrinkage of HDPE onto the HA particles during cooling [66,67,203]. Both HA particle size and their distribution in the HDPE matrix were recognized as important parameters affecting the mechanical behavior of HAPEX™. Namely, smaller HA particles were found to lead to stiffer composites due to general increasing of interfaces between the polymer and the ceramics; furthermore, rigidity of HAPEX™ was found to be proportional to HA volume fraction [187]. Furthermore, coupling agents, e.g., 3-trimethoxysiyl propylmethacrylate for HA and acrylic acid for HDPE might be used to improve bonding (by both chemical adhesion and mechanical coupling) between HA and HDPE [204,205]. Obviously, other types of CaPO_4_ might be used instead of HA in biocomposites with PE [206]. Furthermore, attempts were performed to improve the mechanical properties of HAPEX™ by incorporating other ceramic phases into the polymer matrix, such as PSZ [207] and alumina [208]. For example, a partial replacement of HA filler particles by PSZ particles was found to lead to an increase in the strength and fracture toughness of HA/HDPE biocomposites. The compressive stress, set up by the volume expansion associated with tetragonal to monoclinic phase transformation of PSZ, inhibits or retards the crack propagation within the composite. This results in an enhanced fracture toughness of the HA/ZrO_2_/HDPE biocomposite [207].

Various studies revealed that HAPEX™ attached directly to bones by chemical bonding (a bioactive fixation), rather than forming fibrous encapsulation (a morphological fixation). Initial clinical applications of HAPEX™ came in orbital reconstruction [209] but since 1995, the main uses of this composite have been in the shafts of middle ear implants for the treatment of conductive hearing loss [210,211]. In both applications, HAPEX™ offers the advantage of *in situ* shaping, so a surgeon can make final alterations to optimize the fit of the prosthesis to the bone of a patient and subsequent activity requires only limited mechanical loading with virtually no risk of failure from insufficient tensile strength [66,67,149]. As compared to cortical bones, HA/PE composites have a superior fracture toughness for HA concentrations below ~40% and similar fracture toughness in the 45%–50% range. Their Young’s modulus is in the range of 1–8 GPa, which is quite close to that of bone. The examination of the fracture surfaces revealed that only mechanical bond occurs between HA and PE. Unfortunately, the HA/PE composites are not biodegradable, the available surface area of HA is low and the presence of bioinert PE decreases the ability to bond to bones. Furthermore, HAPEX™ has been designed with a maximized density to increase its strength but the resulting lack of porosity limits the ingrowth of osteoblasts when the implant is placed into the body [23,150]. Further details on HAPEX™ are available elsewhere [66,67]. Except of HAPEX™, other types of both HA/PE [212,213,214,215,216,217,218,219,220] and HA/undisclosed polymer (HA*^nano^* Bone, Promimic AB, Göteborg, Sweden) biocomposites are also known.

Both linear and branched PE was used as a matrix and the biocomposites with the former were found to give a higher modulus [213]. The reinforcing mechanisms in CaPO_4_/polymer formulations have yet to be convincingly disclosed. Generally, if a poor filler choice is made, the polymeric matrix might be affected by the filler through reduction of molecular weight during composite processing, formation of an immobilized shell of polymer around the particles (transcrystallization, surface-induced crystallization or epitaxial growth) and changes in conformation of the polymer due to particle surfaces and inter-particle spacing [66,67]. On the other hand, the reinforcing effect of CaPO_4_ particles might depend on the molding technique employed: a higher orientation of the polymeric matrix was found to result in a higher mechanical performance of the composite [218,219].

Many other blends of CaPO_4_ with various polymers are possible, including rather unusual formulations with dendrimers [221]. Even light-curable CaPO_4_/polymer formulations are known [222]. The list of the appropriate CaPO_4_ is shown in Table 3 (except of MCPM and MCPA – both are too acidic and, therefore, are not biocompatible [16]; nevertheless, to overcome this drawback, they might be mixed with basic compounds, such as HA, TTCP, CaCO_3_, CaO, *etc.*) many biomedically suitable polymers have been listed above. The combination of CaPO_4_ and polymers into biocomposites has a twofold purpose. The desirable mechanical properties of polymers compensate for a poor mechanical behavior of CaPO_4_ bioceramics, while in turn the desirable bioactive properties of CaPO_4_ improve those of polymers, expanding the possible uses of each material within the body [223,224,225,226]. Namely, polymers have been added to CaPO_4_ in order to improve their mechanical strength [223], while CaPO_4_ fillers have been blended with polymers to improve their compressive strength and modulus, in addition to increasing their osteoconductive properties [119,227,228,229,230]. In 1990-s, it was established that with increasing of CaPO_4_ content, both Young’s modulus and bioactivity of the biocomposites generally increased, while the ductility decreased [23]. However, the later investigations revealed that the mechanical properties of CaPO_4_/polymer biocomposites were not so straightforward: the strength was found to decrease with increasing the CaPO_4_ content in such biocomposites [231]. Nevertheless, biocompatibility of such biocomposites is enhanced because CaPO_4_ fillers induce an increased initial flash spread of serum proteins compared to the more hydrophobic polymer surfaces [232]. What’s more, experimental results of these biocomposites indicated favorable cell-material interactions with increased cell activities as compared to each polymer alone [225]. Furthermore, such formulations can provide a sustained release of calcium and orthophosphate ions into the milieus, which is important for mineralized tissue regeneration [224]. Indeed, a combination of two different materials draws on the advantages of each one to create a superior biocomposite with respect to the materials on their own.

It is logical to assume that the proper biocomposite of a CaPO_4_ (for instance, CDHA) with a bioorganic polymer (for instance, collagen) would yield the physical, chemical and mechanical properties similar to those of human bones. Different ways have been already realized to bring these two components together into biocomposites, like mechanical blending, compounding, ball milling, dispersion of ceramic fillers into a polymer-solvent solution, a melt extrusion of a ceramic/polymer powder mixture, co-precipitation and electrochemical co-deposition [22,37,233,234,235]. Three methods for preparing a homogeneous blend of HA with PLLA were compared [233]. A dry process, consisting in mixing ceramic powder and polymer pellets before a compression-molding step, was used. The second technique was based on the dispersion of ceramic fillers into a polymer-solvent solution. The third method was a melt extrusion of a ceramic/polymer powder mixture. Mixing dry powders led to a ceramic particle network around the polymer pellets, whereas the solvent and melt methods also produced a homogeneous dispersion of HA in the matrix. The main drawback of the solvent casting method is the risk of potentially toxic organic solvent residues. The melt extrusion method was shown to be a good way to prepare homogeneous ceramic/polymer blends [233].

Besides, there is *in situ* formation, which involves either synthesizing the reinforcement inside a preformed matrix material or synthesizing the matrix material around the reinforcement [37,236,237]. This is one of the most attractive routes, since it avoids extensive particle agglomeration. For example, several papers have reported *in situ* formation technique to produce various composites of CaPO_4_ with carbon nanotubes [238,239,240,241]. Other examples comprise using amino acid-capped gold nano-sized particles as scaffolds to grow CDHA [242] and preparation of nano-sized HA/PA biocomposites [243,244]. In certain cases, a mechano-chemical route [245,246], emulsions [247,248,249,250,251,252], freeze-drying [253,254] and freeze-thawing techniques [255] or gel-templated mineralization [256] might be applied to produce CaPO_4_-based biocomposites. Various fabrication procedures are well described elsewhere [22,37,233], where the interested readers are referred.

**Figure 1:**
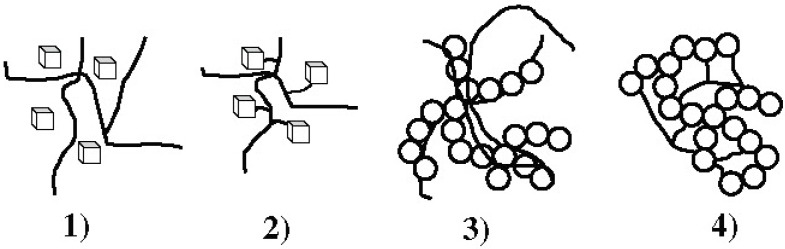
Four types of mutual arrangements of nano-sized particles to a polymer chain: (**1**) inorganic particles embedded in an inorganic polymer, (**2**) incorporation of particles by bonding to the polymer backbone, (**3**) interpenetrating network with chemical bonds, (**4**) inorganic-organic hybrid polymer. Reprinted from Ref. [257] with permission.

The interfacial bonding between the phases is an important issue of any CaPO_4_/polymer biocomposite. Four types of mutual arrangements of nanodimensional particles to polymer chains have been classified by Kickelbick (Figure 1): (1) inorganic particles embedded in inorganic polymer, (2) incorporation of particles by bonding to the polymer backbone, (3) an interpenetrating network with chemical bonds, (4) an inorganic-organic hybrid polymer [257]. If adhesion among the phases is poor, the mechanical properties of a biocomposite suffer. To solve the problem, various approaches have been already introduced. For example, a diisocyanate coupling agent was used to bind PEG/PBT (Polyactive™) block copolymers to HA filler particles. Using surface-modified HA particles as a filler in a PEG/PBT matrix significantly improved the elastic modulus and strength of the polymer as compared to the polymers filled with ungrafted HA [228,258]. Another group used processing conditions to achieve a better adhesion of the filler to the matrix by pressing blends of varying PLLA and HA content at different temperatures and pressures [259]. The researchers found that maximum compressive strength was achieved at ~15 wt % of PLLA. By using blends with 20 wt % of PLLA, the authors also established that increasing the pressing temperature and pressure improved the mechanical properties. The former was explained by decrease in viscosity of the PLLA associated with a temperature increase, hence leading to improved wettability of HA particles. The latter was explained by increased compaction and penetration of pores at higher pressure, in conjunction with a greater fluidity of the polymer at higher temperatures. The combination of high pressures and temperatures was found to decrease porosity and guarantee a close apposition of a polymer to the particles, thereby improving the compressive strength [223] and fracture energy [260] of the biocomposites. The PLLA/HA biocomposites scaffolds were found to improve cell survival over plain PLLA scaffolds [261].

It is also possible to introduce porosity into CaPO_4_-based biocomposites, which is advantageous for most applications as bone substitution material. The porosity facilitates migration of osteoblasts from surrounding bones to the implant site. Various material processing strategies to prepare composite scaffolds with interconnected porosity comprise thermally induced phase separation, solvent casting and particle leaching, solid freeform fabrication techniques, microsphere sintering and coating [123,262,263,264,265]. A supercritical gas foaming technique might be used as well [233,266,267].

#### 4.1.1. Apatite-Based Formulations

A biological apatite is known to be the major inorganic phase of mammalian calcified tissues [14,15]. Consequently, CDHA, HA, carbonateapatite (both with and without dopants) and, occasionally, FA have been applied to prepare biocomposites with other compounds, usually with the aim to improve the bioactivity. For example, polysulfone composed with HA can be used as a starting material for long-term implants [268,269,270]. Retrieved *in vivo*, HA/polysulfone biocomposite coated samples from rabbit distal femurs demonstrated direct bone apposition to the coatings, as compared to the fibrous encapsulation that occurred when uncoated samples were used [268]. The resorption time of such biocomposites is a very important factor, which depends on polymer’s microstructure and the presence of modifying phases [269].

Various apatite-containing biocomposites with PVA [255,271,272,273,274,275], PVAP [276] and several other polymeric components [277,278,279,280,281,282,283,284,285,286] have been already developed. Namely, PVA/CDHA biocomposite blocks were prepared by precipitation of CDHA in aqueous solutions of PVA [255]. An artificial cornea consisted of a porous nano-sized HA/PVA hydrogel skirt and a transparent center of PVA hydrogel has been prepared as well. The results displayed a good biocompatibility and interlocking between artificial cornea and host tissues [271,272]. PVAP has been chosen as a polymer matrix, because its phosphate groups can act as a coupling/anchoring agent, which has a higher affinity toward the HA surface [276]. Greish and Brown developed HA/Ca poly(vinyl phosphonate) biocomposites [280,281,282]. A template-driven nucleation and mineral growth process for the high-affinity integration of CDHA with PHEMA hydrogel scaffold has been developed as well [285].

PEEK [167,168,287,288,289,290,291,292,293] and high impact polystyrene [294,295] were also applied to create biocomposites with HA having a potential for clinical use in load bearing applications. The study on reinforcing PEEK with thermally sprayed HA particles revealed that the mechanical properties increased monotonically with the reinforcement concentration, with a maximum value in the study of ~40% volume fraction of HA particles [287,288,289]. The reported ranges of stiffness within 2.8–16.0 GPa and strength within 45.5–69 MPa exceeded the lower values for human bone (7–30 GPa and 50–150 MPa, respectively) [288]. Modeling of the mechanical behavior of HA/PEEK biocomposites is available elsewhere [290].

Biodegradable poly(α-hydroxyesters) are well established in clinical medicine. Currently, they provide with a good choice when a suitable polymeric filler material is sought. For example, HA/PLGA formulations were developed which appeared to possess a cellular-compatibility suitable for bone tissue regeneration [296,297,298,299,300,301,302,303,304]. Zhang and Ma seeded highly porous PLLA foams with HA particles in order to improve the osteoconductivity of polymer scaffolds for bone tissue engineering [227]. They pointed out that hydration of the foams prior to incubation in simulated body fluid increased the amount of carbonated CDHA material due to an increase of COOH and OH groups on the polymer surface, which apparently acted as nucleation sites for apatite. The mechanical properties of PLA/CaPO_4_ biocomposites fabricated using different techniques, as well as the results of *in vitro* and *in vivo* experiments with them are available in literature [300].

On their own, poly(α-hydroxyesters), such as PGA and PLA, are known to degrade to acidic products (glycolic and lactic acids, respectively) that both catalyze polymer degradation and cause inflammatory reactions of the surrounding tissues [305]. Thus, in biocomposites of poly(α-hydroxyesters) with CaPO_4_, the presence of slightly basic compounds (HA, TTCP) to some extent neutralizes the acid molecules, provides with a weak pH-buffering effect at the polymer surface and, therefore, more or less compensates these drawbacks [119,300,306,307,308]. However, additives of even more basic chemicals (e.g., CaO, CaCO_3_) might be necessary [123,308,309,310]. Extensive cell culture experiments on pH-stabilized composites of PGA and carbonateapatite were reported, which afterwards were supported by extensive *in vitro* pH-studies [311]. A consequent development of this approach has led to designing of functionally graded composite skull implants consisting of polylactides, carbonateapatite and CaCO_3_ [312,313]. Besides the pH-buffering effect, inclusion of CaPO_4_ was found to modify both surface and bulk properties of the biodegradable poly(α-hydroxyesters) by increasing the hydrophilicity and water absorption of the polymer matrix, thus altering the scaffold degradation kinetics. For example, polymer biocomposites filled with HA particles was found to hydrolyze homogeneously due to water penetrating into interfacial regions [314].

**Figure 2:**
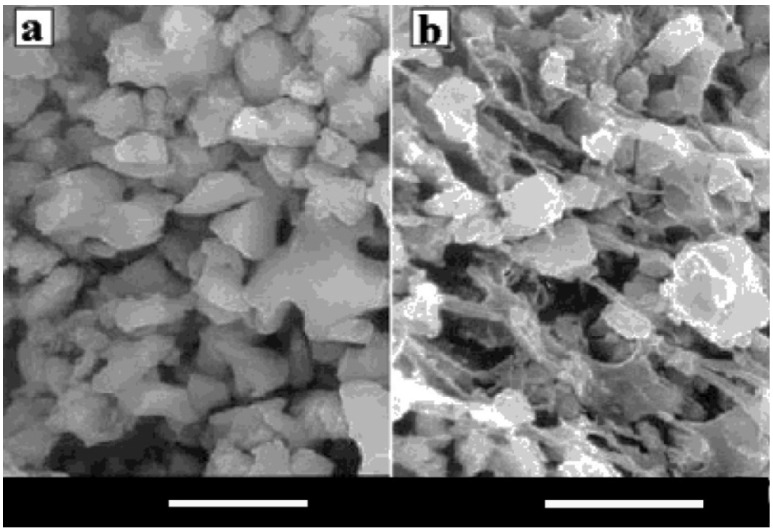
SEM micrographs of (**a**) α-Tricalcium phosphate (α-TCP) compact; (**b**) α-TCP/PLGA biocomposite (bars = 5 µm). Reprinted from Ref. [322] with permission.

Biocomposites of poly(α-hydroxyesters) with CaPO_4_ are prepared mainly by incorporating the inorganic phase into a polymeric solution, followed by drying under vacuum. The resulting solid biocomposites might be shaped using different processing techniques. One can also prepare these biocomposites by mixing HA particles with L-lactide prior the polymerization [306] or by a combination of slip-casting technique and hot pressing [315]; however, other production techniques are known [300,302,316]. Addition of a surfactant (surface active agent) might be useful to keep the suspension homogeneity [317]. Furthermore, HA/PLA [248,249] and HA/PLGA [250] microspheres might be prepared by a microemulsion technique. More complex formulations, such as carbonated-FA/PLA [318] and PLGA/carbon nanotubes/HA [319], are also known. An interesting list of references, assigned to the different ways of preparing HA/poly(α-hydroxyesters) biodegradable composites, might be found in publications by Durucan and Brown [320,321,322]. The authors prepared CDHA/PLA and CDHA/PLGA biocomposites by solvent casting technique with a subsequent hydrolysis of α-TCP to CDHA in aqueous solutions. The presence of both polymers was found to inhibit α-TCP hydrolysis, if compared with that of single-phase α-TCP; what is more, the inhibiting effect of PLA exceeded that of PLGA [320,321,322]. The physical interactions between CaPO_4_ and poly(α-hydroxyesters) might be easily seen in Figure 2 [322]. Another set of good pictures might be found in Ref. [51]. Nevertheless, it should not be forgotten that typically non-melt based routes lead to development of composites with lower mechanical performance and many times require the use of toxic solvents and intensive hand labor [125].

The mechanical properties of poly(α-hydroxyesters) could be substantially improved by addition of CaPO_4_ [323,324]. Namely, CDHA/PLLA biocomposites of very high mechanical properties were developed [119] and fixation tools (screws and plates) made of these composites were manufactured and tested. These fixation tools revealed an easy handling and shaping according to the implant site geometry, total resorbability, good ability to bond directly to the bone tissue without interposed fibrous tissue, osteoconductivity, biocompatibility and high stiffness retainable for the period necessary to achieve bone union [314,316]. The initial bending strength of ~280 MPa exceeded that of cortical bone (120–210 MPa), while the modulus was as high as 12 GPa [119]. The strength could be maintained above 200 MPa up to 25 weeks in phosphate-buffered saline solution. Such biocomposites could be obtained by precipitation from PLLA/dichloromethane solutions, in which small particles of CaPO_4_ were distributed [118,325]. Porous scaffolds of PDLLA + HA [267,326,327] and PLGA + HA [328] have been manufactured as well. Upon implantation into rabbit femora, a newly formed bone was observed and biodegradation was significantly enhanced if compared to single-phase HA bioceramics. This might be due to a local release of lactic acid, which in turn dissolves HA. In other studies, PLA and PGA fibers were combined with porous HA scaffolds. Such reinforcement did not hinder bone ingrowth into the implants, which supported further development of such biocomposites as bone graft substitutes [29,300,301].

Blends (named as SEVA-C) of EVOH with starch filled with 10–30 wt % HA have been fabricated to yield biocomposites with modulus up to ~7 GPa with a 30% HA loading [329,330,331,332,333]. The incorporation of bioactive fillers such as HA into SEVA-C aimed to assure the bioactive behavior of the composite and to provide the necessary stiffness within the typical range of human cortical bone properties. These biocomposites exhibited a strong *in vitro* bioactivity that was supported by the polymer’s water-uptake capability [334]. However, the reinforcement of SEVA-C by HA particles was found to affect the rheological behavior of the blend. A degradation model of these biocomposites has been developed [335].

Higher homologues poly(3-hydroxybutyrate), 3-PHB, and poly(3-hydroxyvalerate) show almost no biodegradation. Nevertheless, biocomposites of these polymers with CaPO_4_ showed a good biocompatibility both *in vitro* and *in vivo* [336,337,338,339,340]. Both bioactivity and mechanical properties of these biocomposites can be tailored by varying the volume percentage of CaPO_4_. Similarly, biocomposites of PHBHV with both HA and amorphous carbonated apatite (almost ACP) appeared to have a promising potential for repair and replacement of damaged bones [341,342,343,344].

Along this line, PCL is used as a slowly biodegradable but well biocompatible polymer. PCL/HA and PCL/CDHA biocomposites have been already discussed as suitable materials for substitution, regeneration and repair of bone tissues [262,345,346,347,348,349,350,351,352]. For example, biocomposites were obtained by infiltration of ε-caprolactone monomer into porous apatite blocks and *in situ* polymerization [346]. The composites were found to be biodegradable and might be applied as cancellous or trabecular bone replacement material or for a cartilage regeneration. Both the mechanical performance and biocompatibility in osteoblast cell culture of PCL were shown to be strongly increased when HA was added [353]. Several preparation techniques of PCL/HA biocomposites are known [262,349]. For example, to make biocomposite fibers of PCL with nanodimensional HA, the desired amount of nanodimensional HA powder was dispersed in a solvent using magnetic stirrer followed by ultrasonication for 30 min. Then, PCL was dissolved in this suspension, followed by the solvent evaporation [354]. The opposite preparation order is also possible: PCL was initially dissolved in chloroform at room temperature (7%–10% w/v), then HA (~10 µm particle size) was suspended in the solution, sonicated for 60 s, followed by the solvent evaporation [355] or salt-leaching [356]. The mechanical properties obtained by this technique were about one-third that of trabecular bone. In a comparative study, PCL and biological apatite were mixed in the ratio 19:1 in an extruder [357]. At the end of the preparation, the mixture was cooled in an atmosphere of nitrogen. The authors observed that the presence of biological apatite improved the modulus while concurrently increasing the hydrophilicity of the polymeric substrate. Besides, an increase in apatite concentration was found to increase both the modulus and yield stress of the composite, which indicated to good interfacial interactions between the biological apatite and PCL. It was also observed that the presence of biological apatite stimulated osteoblasts attachment to the biomaterial and cell proliferation [357]. In another study, a PCL/HA biocomposite was prepared by blending in melt form at 120 °C until the torque reached equilibrium in the rheometer that was attached to the blender [358]. Then the sample was compression molded and cut into specimens of appropriate size for testing. It was observed that the composite containing 20 wt % HA had the highest strength [358]. However, a direct grafting of PCL on the surface of HA particles seems to be the most interesting preparation technique [345]. In another study, HA porous scaffolds were coated by a PCL/HA composite coating [359]. In this system, PCL, as a coating component, was able to improve the brittleness and low strength of the HA scaffolds, while the particles in the coating were to improve the osteoconductivity and bioactivity of the coating layer. More complex formulations, such as PDLLA/PCL/HA [360], PLLA/PCL/HA [361], FA-HA/PCL [362], magnetic PCL/Fe-doped HA [363] and supramolecular PCL/functionalized HA [364,365] biocomposites, have been prepared as well. Further details on both the PCL/HA biocomposites and the processing methodologies thereof might be found elsewhere [262,349].

**Figure 3:**
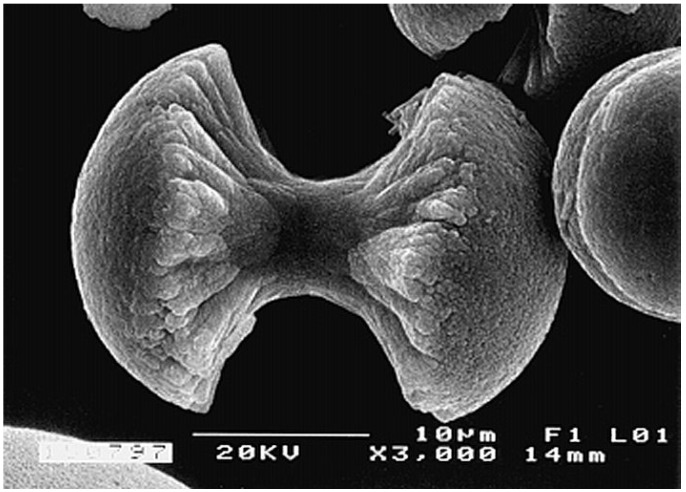
A biomimetically grown aggregate of fluorapatite (FA) that was crystallized in a gelatin matrix. Its shape can be explained and simulated by a fractal growth mechanism. Scale bar: 10 μm. Reprinted from Ref. [366] with permission.

An interesting phenomenon of fractal growth of FA/gelatin composite crystals (Figure 3) was achieved by diffusion of calcium- and orthophosphate + fluoride-containing solutions from the opposite sides into a tube filled with a gelatin gel [366,367,368,369,370,371,372]. The reasons of this phenomenon are not quite clear yet. Other types of CaPO_4_-based composites, based on DCPD and OCP, were grown by the similar technique in an iota-carrageenan gel [373]. Up to now, nothing has yet been reported on a possible biomedical application of such unusual structural composites.

To finalize this section, one should note that there are polymers possessing shape-memory properties [374] and, therefore, CaPO_4_-based composites with such polymers appear to have the shape-memory properties as well [375,376,377].

#### 4.1.2. TCP-Based Formulations

Both α-TCP and β-TCP have a higher solubility than HA (Table 3). Besides, they are faster resorbed *in vivo* (however, there are some reports about a lack of TCP biodegradation after implantation in calvarial defects [378]). Therefore, α-TCP and β-TCP were widely used instead of apatites to prepare completely biodegradable biocomposites [379,380,381,382,383,384,385,386,387,388,389,390,391,392,393,394,395,396,397,398,399]. For example, a biodegradable and osteoconductive biocomposite made of β-TCP particles and gelatin was proposed [383]. This material was tested *in vivo* with good results. It was found to be biocompatible, osteoconductive and biodegradable with no need for a second surgical operation to remove the device after healing occurred. Both herbal extracts [384] and K_2_HPO_4_ [385] might be added to this formulation. Another research group prepared biocomposites of crosslinked gelatin with β-TCP and both a good biocompatibility and bone formation upon subcutaneous implantation in rats were found [386]. Yang *et al.*, [390] extended this to porous (porosity ~75%) β-TCP/gelatin biocomposites those also contained BMP-4. Furthermore, cell-compatible and possessive some osteoinductive properties porous β-TCP/alginate-gelatin hybrid scaffolds were prepared and successfully tested *in vitro* [387]. In addition, the CaPO_4_ fillers were found to have a reinforcing effect [400]. More to the point, biocomposites of β-TCP with PLLA [325,379,380,381] and PLGC [382] were prepared. Although β-TCP was able to counter the acidic degradation of the polyester to some extent, it did not prevent a pH drop down to ~6. Nevertheless, implantation of this biocomposite in beagles’ mandibular bones was successful [382]. α-TCP/gelatin formulations are known as well [393].

Based on a self-reinforcement concept, biocomposites of TCP with polylactides were prepared and studied using conventional mechanical testing [401]. Resorbable scaffolds were fabricated from such biocomposites [402]. Chitosan was also used as the matrix for the incorporation of β-TCP by a solid/liquid phase separation of the polymer solution and subsequent sublimation of the solvent. Due to complexation of the functional groups of chitosan with calcium ions of β-TCP, these biocomposites had high compressive modulus and strength [403]. PCL/β-TCP biocomposites were developed in other studies [404,405,406,407,408] and their *in vitro* degradation behavior was systematically monitored by immersion in simulated body fluid at 37 °C [406]. To extend this topic further, PCL/β-TCP biocomposites might be loaded by drugs [407].

An *in vitro* study with primary rat calvarial osteoblasts showed an increased cellular activity in the BMP-loaded samples [390]. Other researchers investigated BMP-2-loaded porous β-TCP/gelatin biocomposites (porosity ~ 95%, average pore size 180–200 µm) [409] and confirmed the precious study. A long-term implantation study of PDLLA/α-TCP composites in a loaded sheep implant model showed good results after 12 months but a strong osteolytic reaction after 24 months. This was ascribed to the almost complete dissolution of α-TCP to this time and an adverse reaction of the remaining PDLLA [410].

More complex CaPO_4_-based formulations are known as well. For example, there is a biocomposite consisting of three interpenetrating networks: TCP, CDHA and PLGA [411]. Firstly, a porous TCP network was produced by coating a PU foam by hydrolysable α-TCP slurry. Then, a CDHA network was derived from self-setting CaPO_4_ formulations filled in the porous TCP network. Finally, the remaining open pore network in the CDHA/α-TCP structures was infiltrated with PLGA. This biocomposite consists of three phases with different degradation behavior. It was postulated that bone would grow on the fastest degrading network of PLGA, while the remaining CaPO_4_ phases would remain intact thus maintaining their geometry and load bearing capability [411].

#### 4.1.3. Formulations Based on Other Types of CaPO_4_

The number of research publications devoted to formulations based on other types of CaPO_4_ is substantially lesser than those devoted to apatites and TCP. Biphasic calcium phosphate (BCP), which is a solid composite of HA and β-TCP (however, similar formulations of HA and α-TCP, as well as of α-TCP and β-TCP are known, as well [412]) appears to be most popular among the remaining types of CaPO_4_. For example, collagen coated BCP ceramics was studied and the biocompatibility towards osteoblasts was found to increase upon coating with collagen [413]. Another research group created porous PDLLA/BCP scaffolds and coated them with a hydrophilic PEG/vancomycin composite for both drug delivery purposes and surface modification [414]. More to the point, both PLGA/BCP [415,416] and PLLA/BCP [417] biocomposites were fabricated and their cytotoxicity and fibroblast properties were found to be acceptable for natural bone tissue reparation, filling and augmentation [418,419]. Besides, PCL/BCP [420,421], PTMC/BCP [422] and gelatin/BCP [423,424] biocomposites are known as well.

A choice of DCPD-based biocomposites of DCPD, albumin and duplex DNA was prepared by water/oil/water interfacial reaction method [247]. Core-shell type DCPD/chitosan biocomposite fibers were prepared by a wet spinning method in another study [425]. The energy-dispersive X-ray spectroscopy analysis indicated that Ca and P atoms were mainly distributed on the outer layer of the composite fibers; however, a little amount of P atoms remained inside the fibers. This indicated that the composite fibers formed a unique core-shell structure with shell of CaPO_4_ and core of chitosan [425]. A similar formulation was prepared for further applications in self-setting biocomposites [426]. DCPA/BSA biocomposites were synthesized through the co-precipitation of BSA on the nanodimensional particles of DCPA performed in ethanol [427]. Nanodimensional DCPA was synthesized and incorporated into dental resins to form dental biocomposites [428,429,430,431]. Although, this is beyond the biomedical subject, it is interesting to mention that some DCPD/polymer composites could be used as proton conductors in battery devices [432,433]. Nothing has been reported on their biocompatibility but, perhaps, sometime the improved formulations will be used to fabricate biocompatible batteries for implantable electronic devices.

Various ACP-based biocomposites and hybrid formulations for dental applications have been developed [434,435]. Besides, several ACP-based formulations were investigated as potential biocomposites for bone grafting [344,436,437,438] and drug delivery [439,440]. Namely, ACP/PPF biocomposites were prepared by *in situ* precipitation [437], while PHB/carbonated ACP and PHBHV/carbonated ACP biocomposites appeared to be well suited as slowly biodegradable bone substitution material [344].

Finally, information on the biomedically relevant OCP-based formulations might be found in a topical review [441].

### 4.2. Self-Setting Formulations

Inorganic self-setting CaPO_4_ formulations (cements) hardening in the body were introduced in the early 1980-s. Since then, they have been broadly studied and many formulations have been proposed. These formulations set and harden due to various chemical interactions among CaPO_4_ that finally lead to formation of a monolithic body consisting of either CHDA or DCPD with possible admixtures of other phases. Unfortunately, having a ceramic nature, the self-setting CaPO_4_ formulations are brittle after hardening and the setting time is sometimes unsuitable for clinical procedures [442]. Therefore, to improve their properties, various attempts have been performed to transform them into biocomposites, e.g., by adding hydroxycarboxylic acids [443,444,445], gelatin [389,446,447,448,449], osteocalcin/collagen [450], alginate [451], chitin [452], chitosan [453], silk fibroin [454], silanized HPMC [455], bioactive glass [456], magnetic nanoparticles [457], *etc.* More to the point, various reinforcement additives of different shapes and nature are widely used to improve the mechanical properties of the self-setting formulations [458,459]. Even carbon nanotubes are used for this purpose [460,461]. Although the biomaterials community does not use this term, a substantial amount of the reinforced formulations might be defined as CaPO_4_-based concretes [442]. The idea behind the concretes is simple: if a strong filler is present in the matrix, it might stop crack propagation.

Various apatite-containing formulations based on PMMA [462,463,464,465,466,467,468,469,470] and polyethylmethacrylate [471,472] have been already developed. Such biocomposites might be prepared by dispersion of apatite powder into a viscous fluid of the polymer [473] and used for drug delivery purposes [474]. When the mechanical properties of the concretes composed of PMMA matrix and HA particles of various sizes were tested, the tensile results showed that strength was independent on particle sizes. After immersion into Ringer´s solution, the tensile strength was not altered whereas the fatigue properties were significantly reduced. The biocompatibility of PMMA/HA formulations was tested *in vivo* and enhanced osteogenic properties of the implants compared to single-phase PMMA were observed [463,464,465,466]. It was shown that not only the mechanical properties of PMMA were improved but the osteoblast response of PMMA was also enhanced with addition of HA [464]. Thereby, by adding of CaPO_4_, a non-biodegradable PMMA was made more bioactive and osteoconductive, yielding a well-processible biocomposite concrete. As a drawback, the PMMA/HA formulations possess a low flexural, compressive and tensile strength.

Biocomposites made from CaPO_4_ and various types of resins appeared to possess comparable mechanical and biological properties to typical PMMA cement, leading to potential uses for implant fixation [170,172,475,476,477]. To improve the mechanical properties of the self-setting CaPO_4_-based formulations and stabilize them at the implant site, various researchers have resorted to formulations that set *in situ*, primarily through crosslinking reactions of the polymeric matrix. For example, TTCP was reacted with PAA, forming a crosslinked CDHA/calcium polyacrylate biocomposite [478]. In aqueous solutions, TTCP hydrolyzes to CDHA [16] and the liberated calcium cations react with PAA, forming the crosslinked network [478]. Reed *et al.*, synthesized a dicarboxy polyphosphazene that can be crosslinked by calcium cations and CDHA/polyphosphazene biocomposites with a compressive strength ~10 MPa and of ~65% porosity were prepared as a result [479]. To mimic PMMA cements, PFF/β-TCP biocomposites were prepared with addition of vinyl monomer to crosslink PPF. As a result, quick setting and degradable formulations with a low heat output and compressive strengths in the range of 1–12 MPa were prepared by varying the molecular weight of PPF, as well as the contents of the monomer, β-TCP, initiator and NaCl, as a porogen [480,481]. An acrylic formulation with Sr-containing HA as a filler [100], an injectable polydimethylsiloxane/HA formulation [482], biocomposites consisting of PLGA microspheres and self-setting CaPO_4_-based formulations [483,484], as well as hybrid formulations of chitosan oligosaccharide/gelatin/CaPO_4_ [485] were prepared as well.

In order to improve the mechanical properties of self-setting formulations, numerous researchers blended various polymers to them. For example, gelatin might be added to self-setting formulations, primarily to stabilize the paste in aqueous solution before it develops adequate rigidity and, secondly, to improve the compressive strength [389,446,486]. Adding rod-like fillers to the self-setting formulations also caused an improvement in the mechanical properties [486]. For example, PAA and PVA were successfully used to improve the mechanical properties of a TTCP + DCPD formulation but, unfortunately, with an inevitable and unacceptable reduction of both workability and setting time [487,488]. Similar findings were reported in the presence of sodium alginate and sodium polyacrylate [489]. Other polymers, such as polyphosphazene might be used as well [490,491,492]. Other examples of polymer/CaPO_4_ self-setting formulations might be found elsewhere [493,494]. Metallic wires might be used as reinforcements as well [495].

Porous CaPO_4_ scaffolds with interconnected macropores (~1 mm), micropores (~5 μm) and of high porosity (~80%) were prepared by coating PU foams with a TTCP + DCPA formulation, followed by firing at 1200 °C. In order to improve the mechanical properties of the scaffolds, the open micropores of the struts were then infiltrated by a PLGA solution to achieve an interpenetrating bioactive ceramic/biodegradable polymer composite structure. The PLGA filled struts were further coated with a 58S bioactive glass/PLGA composite coating. The obtained complex porous biocomposites could be used as tissue engineering scaffolds for low-load bearing applications [496]. A more complicated construction, in which the PLGA macroporous phase has been reinforced with a bioresorbable TTCP + DCPA formulation, followed by surface coating of the entire construct by a non-stoichiomentic CDHA layer, has been designed as well [497]. In one more study, α-TCP and poly(ethylenephosphate) sodium salt-coated PLGA microparticles were mixed with castor oil and water to form an oil-in-water emulsion. The resulting emulsion spontaneously set in a humidified atmosphere at ambient temperature. The PLGA particles incorporated within the set formulations were hydrolytically degraded, which resulted in the formation of an interconnected microporosity [498].

A porosity level of 42%–80% was introduced into self-setting CaPO_4_/chitosan biocomposites by addition of the water-soluble mannitol [499]. Chitosan significantly improved the mechanical strength of the entire biocomposite [500]. A similar approach was used by other researchers who studied the effect of the addition of PLGA microparticles [501,502,503,504] (which can also be loaded with drugs or growth factors [505,506,507]) to self-setting CaPO_4_ formulations. These biocomposites were implanted into cranial defects of rats and a content of ~ 30 wt. % of the microparticles was found to give the best results [501], while the addition of a growth factor to the biocomposites significantly increased bone contact at 2 weeks and enhanced new bone formation at 8 weeks [507]. The *in vivo* rabbit femur implant tests showed that self-setting PLGA/CaPO_4_ formulations exhibited outstanding biocompatibility and bioactivity, as well as a better osteoconduction and degradability than self-setting formulations consisted solely from CaPO_4_ [502].

To finalize this topic, one should mention that CaPO_4_ might be added into self-setting calcium sulfate (plaster of Paris) formulations to improve their osteoconductivity [508,509]. Similarly, calcium silicates could be added to the self-setting CaPO_4_ formulations [510,511,512,513].

### 4.3. Formulations Based on Nanodimensional CaPO_4_ and Nanodimensional Biocomposites

Nanodimensional and nanophasic materials are the materials that have particles or grain sizes less than 100 nm, respectively. However, before proceeding any further, one should clearly differentiate between the nanodimensional composites and the composites containing nanodimensional particles. The former might be any type of composites but disintegrated to particles with dimensions <100 nm, while the latter consist of two or more materials, in which at least one of the materials is of a nanometer-scale. Nevertheless, both options combine the properties of both components, thus broadening their functionality beyond the pure materials.

Nanodimensional and nanophasic materials have different mechanical and optical properties if compared to the large grained materials of the same chemical composition. Namely, they possess the unique surface properties, such as an increased number of atoms, grain boundaries and defects at the surface, huge surface area and altered electronic structure, if compared to the conventional micron-sized materials. For example, nanodimensional HA (size ~67 nm) has a higher surface roughness of 17 nm if compared to 10 nm for the conventional submicron size HA (~180 nm), while the contact angles (a quantitative measure of the wetting of a solid by a liquid) are significantly lower for nanodimensional HA (6.1) if compared to the conventional HA (11.51). Additionally, the diameter of individual pores in nanodimensional HA compacts is five-times smaller (pore diameter ~6.6 Å) than that in the conventional grain-sized HA compacts (pore diameter within 19.8–31.0 Å) [514]. Besides, nanodimensional CaPO_4_ promote osteoblast cells adhesion, differentiation and proliferation, osteointegration and deposition of calcium containing minerals on its surface better than microcrystalline ones; thus enhancing formation of a new bone tissue within a short period [515,516,517]. More to the point, nanodimensional HA was found to cause apoptosis of the leukemia P388 cells [518].

Natural bones and teeth are hierarchical biocomposites of biological origin based on nanodimensional compounds because they consists of nano-sized blade-like crystals of biological apatite grown in intimate contact with the organic matrix rich in bioorganic fibers and organized in complicated hierarchical structures. Given the fact that the major bioorganic phase of bones is collagen, *i.e.*, a natural polymer (Table 1), it is obvious that biocomposites of nanodimensional CaPO_4_ with biodegradable polymers should be advantageous as bone grafting materials. In such biocomposites, the inorganic phase would be responsible for the mechanical strength (hardness) and bioactivity, while the polymeric phase would provide the elasticity. In addition, the solubility of CaPO_4_ depends on their crystallite size (smaller crystals have a higher solubility) and on their carbonate content (higher carbonate content increases the solubility) [519]. To the author’s best knowledge, among CaPO_4_ listed in Table 3, before very recently only apatites (CDHA, HA and, perhaps, FA) have been available in nanodimensional state. However, recently, nano-sized DCPA [428,429,430] and nano-sized MCPM [520] have been synthesized and applied to prepare biocomposites with strong ionic release to combat tooth caries. Presumably, all CaPO_4_ from Table 3 might be manufactured in nanodimensional and/or nanocrystalline state; however not all of them have been prepared yet [517].

**Figure 4:**
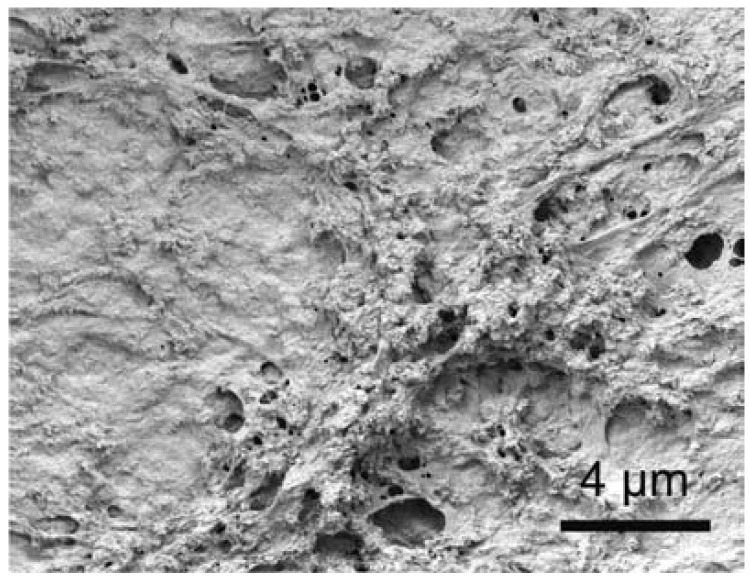
Scanning electron microscopy image of reconstituted mineralized collagen I fibrils. An example of an organic-inorganic nanostructural composite, mimicking the extracellular matrix of bone tissue on the nanometer scale. Reprinted from Ref. [637] with permission.

A number of investigations have been conducted recently to determine the mineralization, biocompatibility and mechanical properties of the biocomposites based on various (bio)polymers and nanodimensional CaPO_4_ (mainly, HA). Unfortunately, in the majority of the already published papers it often remained unclear whether “nanodimensional HA”, in fact, represented the nanodimensional stoichiometric HA or a nanodimensional non-stoichiometric CDHA; therefore, no differentiation is possible between them. These studies covered biocomposites with PGA [521], PLA [176,267,522,523,524,525,526,527,528,529,530,531,532,533] and its copolymers with PGA [534,535,536] and PCL [537], collagen [538,539,540,541,542,543,544,545,546,547,548], collagen + PLA [549,550,551,552,553,554], collagen + PVA [555], collagen + alginate [556,557], alginate [558], gelatin [559,560,561,562,563,564], PPF [565,566,567], PA [243,244,568,569,570,571,572,573,574,575], PVA [271,272,576,577,578], PVAP [276], poly(ethylene*-co-*acrylic) acid [579,580], chitosan [581,582,583,584,585,586,587,588,589] and its derivatives [590], konjac glucomannan + chitosan [591], chitosan + gelatin [592], xanthan [593], PHEMA + PCL [594], PCL [317,354,595,596,597], PU [598], cellulose [41,42,599,600,601,602], Ti [602,603,604], ZrO_2_ [605,606,607], glasses [608,609], metals and alloys [610,611] and many other biocompatible hybrid formulations [215,256,264,270,342,612,613,614,615,616,617,618,619,620,621,622,623,624,625,626,627,628,629,630,631]. Furthermore, each from the aforementioned formulations might be covered by a layer of nanodimensional CaPO_4_, as it was done by Zandi *et al.* [632], who coated a biocomposite of nano-sized rods HA with gelatin by nano-sized HA. Several nanodimensional biocomposites were found to be applicable as carriers for delivery of drugs and growth factors [609,633,634,635], as well as promising vectors with ultrahigh gene loading and transfection efficiency [636]. Data are available on the excellent biocompatibility of such biocomposites [545]. The dispersion state of nano-sized particles appears to be the critical parameter in controlling the mechanical properties of nanodimensional biocomposites, as nano-sized particles always tend to aggregate owing to their high surface energy [342]. A comparison was made of the mechanical properties between biocomposites of PA with the nano-sized and micron-sized HA. The results showed that the bending and tensile strengths of the biocomposite increased with increasing content of nanodimensional HA but decreased with increasing micron-sized HA content [243]. A SEM image of the mineralized collagen fibrils, demonstrating homogeneity of the nanodimensional biocomposite and the close interaction between the mineral phase and the reconstituted collagen fibrils is shown in Figure 4 [637].

Porous (porosity ~85%) biocomposites of nano-sized HA with collagen and PLA have been prepared by precipitation and freeze-drying; these biocomposites did not show a pH drop upon *in vitro* degradation [549,550,551]. They were implanted in the radius of rabbits and showed a high biocompatibility and partial resorption after 12 weeks. Nano-sized HA/сhitosan biocomposites with improved mechanical stability were prepared from HA/сhitosan nano-sized rods [638]. Nano-sized HA/PLLA biocomposites of high porosity (~90%) were prepared using thermally induced phase separation [639]. Besides, nanodimensional HA was used to prepare biocomposites with PAA and the nanostructure of the resulting nano-sized crystals exhibited a core-shell configuration [640,641].

Nanodimensional crystals of HA appeared to be suitable for intra-osseous implantation and offered a potential to formulate enhanced biocomposites for clinical applications [642]. Thus, the biocompatibility of chitosan in osteoblast cell culture was significantly improved by addition of nano-sized HA [643]. Similar finding was valid for nanodimensional HA/PA biocomposites [569]. Further details on nanodimensional biocomposites might be found in excellent reviews [22,644]. More to the point, a more general review on applications of nanodimensional biomaterials in orthopedics is also available [645], where the interested readers are referred.

### 4.4. Biocomposites with Collagen

The main constituent of the bioorganic matrix of bones is type I collagen (Table 1) with molecules about 300 nm in length. The structural and biochemical properties of collagens have been widely investigated and over 25 collagen subtypes have been identified [646,647]. This protein is conducive to crystal formation in the associated inorganic matrix. It is easily degraded and resorbed by the body and allows good attachment to cells. Collagen alone is not effective as an osteoinductive material but it becomes osteoconductive in combination with CaPO_4_ [648]. Both collagen type I and HA were found to enhance osteoblast differentiation [649] but combined together, they were shown to accelerate osteogenesis. However, this tendency is not so straightforward: the data are available that implanted HA/collagen biocomposites enhanced regeneration of calvaria bone defects in young rats but postponed the regeneration of calvaria bone in aged rats [650]. Finally, addition of CaPO_4_ to collagen sheets was found to give a higher stability and an increased resistance to 3D swelling compared to the collagen reference [651]. Therefore, a bone analogue based on these two constituents should possess the remarkable properties.

**Figure 5:**
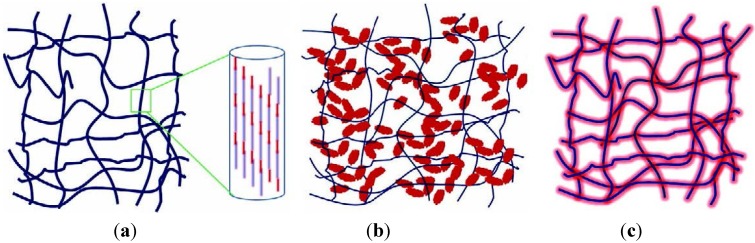
Schematic illustrations of collagen–CaPO_4_ nanocomposites: (**a**) structure of bone: nanodimensional crystals of biological apatite aligned along with the collagen fibrils; linking occurs between the apatite and polar groups on collagen chains in bone; (**b**) nanodimensional crystals of CaPO_4_ physically “trapped” within the collagen matrix; (**c**) nanodimensional crystals of CaPO_4_ deposited on and covered the collagen fibrils surface. Reprinted from Ref. [652] with permission.

The unique characteristics of bones are the spatial orientation between the nanodimentional crystals of biological apatite and collagen macromolecules at the nano-scale, where the crystals (about 50 nm length) are aligned parallel to the collagen fibrils, which is believed to be the source of the mechanical strength of bones (Figure 5a) [652]. The collagen molecules and the crystals of biological apatite assembled into mineralized fibrils are approximately 6 nm in diameter and 300 nm long. Although the complete mechanisms involved in the bone building strategy are still unclear, the strengthening effect of nanodimentional crystals of biological apatite in calcified tissues might be explained by the fact that the collagen matrix is a load transfer medium and thus transfers the load to the intrinsically rigid inorganic crystals [14,15,19,20,21]. Furthermore, the crystals of biological apatite located in between tangled fibrils cross-link the fibers either through a mechanical interlocking or by forming calcium ion bridges, thus increasing deformation resistance of the collagenous fiber network [653].

When CaPO_4_ are combined with collagen in a laboratory, the prepared biocomposites appear to be substantially different from natural bone tissue (Figure 5b,c) due to a lack of real interaction between the two components, *i.e.*, the interactions that are able to modify the intrinsic characteristics of the singular components themselves. The main characteristics of the route, by which the mineralized hard tissues are formed *in vivo*, is that the organic matrix is laid down first and the inorganic reinforcing phase grows within this organic matrix [14,15,20,21]. Although to date, neither the elegance of the biomineral assembly mechanisms nor the intricate composite nano-sized architectures have been duplicated by non-biological methods, the best way to mimic bone is to copy the way it is formed, namely by nucleation and growth of CDHA nano-sized crystals from a supersaturated solution both onto and within the collagen fibrils. Such syntheses were denoted as ‘‘biologically inspired’’ which means they reproduce an ordered pattern and an environment very similar to natural ones [654,655,656]. The biologically inspired biocomposites of collagen and CaPO_4_ (mainly, apatites) for bone substitute have a long history [648,657,658,659,660,661,662,663,664,665,666,667,668,669,670] and started from the pioneering study by Banks *et al.*, who grew CDHA on reconstituted calfskin collagen tapes in 1977 [671], followed by initial medical applications performed by other researchers in 1982 [672,673]. Such combinations were found to be bioactive, osteoconductive, osteoinductive [648,674,675,676] and, in general, artificial grafts manufactured from this type of the biocomposites are likely to behave similarly to bones and be of more use in surgery than those prepared from any other materials. Indeed, data are available on the superiority of CaPO_4_/collagen biocomposite scaffolds over the artificial polymeric and CaPO_4_ bioceramic scaffolds individually [677].

It has been found that CaPO_4_ may be successfully precipitated onto a collagen substrate of whatever form or source [539,671,678,679]. However, adherence of CaPO_4_ crystals to collagen did depend on how much the collagen had been denatured: the more fibrillar the collagen, the greater attachment. Clarke *et al.*, first reported the production of a biocomposite produced by precipitation of DCPD onto a collagen matrix with the aid of phosphorylated amino acids commonly associated with fracture sites [657]. Self-setting CDHA forming formulations (DCPD + TTCP) have been mixed with a collagen suspension, hydrated and allowed to set. CDHA crystals were found to nucleate on the collagen fibril network, giving a material with the mechanical properties weaker than those reported for bone. More to the point, the prepared biocomposites were without the nanostructure similar to that of bone [658,680]. The oriented growth of OCP crystals on collagen was achieved by an experimental device in which Ca^2+^ and PO_4_^3−^ ions diffused into a collagen disc from the opposite directions [680,681,682]. Unfortunately, these experiments were designed to simulate the mechanism of *in vivo* precipitation of biological apatite only; due to this reason, the mechanical properties of the biocomposites were not tested [683].

Conventionally, collagen/CaPO_4_ biocomposites can be prepared by blending or mixing of collagen and CaPO_4_, as well as by biomimetic methods [539,550,634,654,655,656,657,658,668,678,683,684,685,686,687,688,689,690,691,692,693,694]. Tampieri *et al.*, [656] produced and compared artificial bone like tissue apatite/collagen biocomposites prepared by using two different methodologies: (1) dispersion of apatite in a collagen aqueous suspension and then freeze dried and (2) direct nucleation of an apatitic phase on assembling collagen fibrils. Biocomposites obtained using first way were similar to uncalcified natural collagen. The crystallite sizes were not uniform and were often aggregated and randomly distributed into the matrix (Figure 5b), proving that there was no real interaction between apatite and collagen fibers. However, the second method allowed the direct nucleation of nano-sized crystals of apatite on self-assembled collagen fibers. In this case, the two components (CDHA and collagen) exhibited strong interactions (Figure 5c), highlighted by several analysis techniques, which showed a complete analogy of the composite with calcified natural tissue [656]. Other production techniques are also possible. For example, using a polymer-induced liquid-precursor process, collagen/apatite biocomposites mimicking the nanostructure of bones, wherein nano-sized crystals of apatite were embedded within the collagen fibrils, were prepared [693]. More complicated formulations, such as a magnetite enriched HA/collagen [695] and HA/collagen/PVA [696] biocomposites, have been developed as well. Recent investigations revealed that CaPO_4_/collagen formulations might be printed by means of 3D printers [697].

In addition, collagen might be incorporated into various self-setting CaPO_4_-based formulations [658,680,698,699,700,701,702]. Typically, a type I collagen sponge is presoaked in PO_4_^3−^-containing a highly basic aqueous solution and then is immersed into a Ca^2+^-containing solution to allow mineral deposition. Also, collagen I fibers might be dissolved in acetic acid and then this solution is added to phosphoric acid, followed by a neutralization synthesis (performed at 25 °C and solution pH within 9–10) between an aqueous suspension of Ca(OH)_2_ and the H_3_PO_4_/collagen solution [654,655]. To ensure the quality of the final product, it is necessary to control the Ca/P ionic ratio in the reaction solution. One way to do this is to dissolve a commercial CaPO_4_ in an acid; another one is to add Ca^2+^ and PO_4_^3−^ ions in a certain ratio to the solution and after that induce the reaction [703]. Biomimetically, one can achieve an oriented growth of CDHA crystals onto dissolved collagen fibrils in aqueous solutions via a self-organization mechanism [688]. Besides, CDHA crystallization from aqueous solutions might be performed in the presence of a previously dispersed collagen [539]. More to the point, collagen might be first dispersed in an acidic solution, followed by addition of calcium and orthophosphate ions and then co-precipitation of collagen and CDHA might be induced by either increasing the solution pH or adding mixing agents. Although it resulted in biocomposites with poor mechanical properties, pressing of the apatite/collagen mixtures at 40 °C under 200 MPa for several days is also known [704]. Attempts have been performed for a computer simulation of apatite/collagen composite formation process [705]. It is interesting to note, that such biocomposites were found to possess some piezoelectric properties [706].

As the majority of the collagen/HA biocomposites are conventionally processed by anchoring micron-sized HA particles into collagen matrix, it makes quite difficult to obtain a uniform and homogeneous composite graft. Besides, such biocomposites have inadequate mechanical properties; over and above, the proper pore sizes have not been achieved either. Further, microcrystalline HA, which is in contrast to nanocrystalline bone apatite, might take a longer time to be remodeled into a new bone tissue upon the implantation. In addition, some of the biocomposites exhibited very poor mechanical properties, probably due to a lack of strong interfacial bonding between the constituents. Furthermore, in all blended composites, the crystallite sizes of CaPO_4_ were not uniform and the crystals were often aggregated and randomly distributed within a fibrous matrix of collagen. Therefore, no structural similarity to natural bone was obtained and only a compositional similarity to that of natural bone was achieved. The aforementioned data clearly demonstrate that the chemical composition similar to bone is insufficient for manufacturing the proper grafts; both the mechanical properties and mimetic of the bone nanostructure are necessary to function as bone in recipient sites. There is a chance for improving osteointegration by reducing the grain size of HA crystals by activating of ultrafine apatite growth into the matrix. This may lead to enhance the mechanical properties and osteointegration with improved biological and biochemical affinity to the host bone. Besides, the porosity was found to have a positive influence on the ingrowth of the surrounding tissues into the pores of collagen/HA biocomposites [707,708].

Bovine collagen might be mixed with CaPO_4_ and such biocomposites are marketed commercially as bone-graft substitutes those further can be combined with bone marrow aspirated from the iliac crest of the site of the fracture. Bioimplant^®^, Bio-Oss Collagen^®^, Boneject^®^, Collagraft^®^, CollapAn^®^, Healos^®^, Integra Mozaik^®^, and LitAr^®^ are several examples of the commercially available CaPO_4_/collagen grafts for the clinical use [17,22]. Application of these materials was compared with autografts for the management of acute fractures of long bones with defects, which had been stabilized by internal or external fixation [709,710]. These biocomposites are osteogenic, osteoinductive and osteoconductive; however, they lack the structural strength and require a harvest of the patient’s bone marrow. OCP/collagen biocomposites have been investigated [711] and clinically tested [712].

Collagen sponges with an open porosity (30–100 μm) were prepared by a freeze-drying technique and then their surface was coated by a 10-μm layer of biomimetic apatite precipitated from simulated body fluid [713]. The researchers found a good *in vitro* performance with fibroblast cell culture. Other preparation techniques are also possible [714]. Collagen/HA microspheres or gel beads have been prepared in the intention of making injectable bone fillers [715,716,717]. Liao *et al.* succeeded in mimicking the bone structure by blending carbonateapatite with collagen [718]. A similar material (mineralized collagen) was implanted into femur of rats and excellent clinical results were observed after 12 weeks [719]. Collagen/HA biocomposites were prepared and their mechanical performance was increased by cross-linking the collagen fibers with glutaraldehyde [540,541,542]. These biocomposites were tested in rabbits and showed a good biological performance, osteoconductivity and biodegradation. A similar approach was selected to prepare HA/collagen microspheres (diameter ~5 μm) by a water-oil emulsion technique in which the surface was also cross-linked by glutaraldehyde [716]. That material showed a good *in vitro* performance with osteoblast cell culture. A porous bone graft substitute was formed from a nano-sized HA/collagen biocomposite combined with PLA by a freeze-drying method; the resulting material was found to mimic natural bones at several hierarchical levels [550]. Subsequent *in vitro* experiments confirmed a good adhesion, proliferation and migration of osteoblasts into this composite [549]. A further increase in biocompatibility might be achieved by addition of various dopants. For example, to enhance bone substitution, Si-substituted HA/collagen composites have been developed with silicon located preferentially in the collagen phase [541]. Porous (porosity level ~95% with interconnected pores of 50–100 μm) biocomposites of collagen (crosslinked with glutaraldehyde) and β-TCP have been prepared by a freeze-drying technique, followed by sublimation of the solvent; the biocomposites showed a good biocompatibility upon implantation in the rabbit jaw [720].

Biocomposites of CaPO_4_ with collagen were found to be useful for delivery of drugs, growth factors and other important biomolecules [557,660,702,721,722,723]. Namely, an HA/collagen – alginate (20 µL) with the rhBMP-2 (100 µg/mL, 15 µL) showed bone formation throughout the implant 5 weeks after implantation without obvious deformation of the material [557]. Gotterbarm *et al.*, developed a two-layered collagen/β-TCP implant augmented with chondral inductive growth factors for repair of osteochondral defects in the trochlear groove of minipigs. This approach might be a new promising option for the treatment of deep osteochondral defects in joint surgery [722].

To conclude this part, one should note that biocomposites of apatites with collagen are a very hot topic for research and up to now, just a few papers have been devoted to biocomposites of other CaPO_4_ with collagen [685,686,687,722,724,725,726]. These biomaterials mimic natural bones to some extent, while their subsequent biological evaluation suggests that they are readily incorporated into the bone metabolism in a way similar to bone remodeling, instead of acting as permanent implant [550,673]. However, the performance of these biocomposites depends on the source of collagen from which it was processed. Several attempts have been made to simulate the collagen-HA interfacial behavior in real bone by means of crosslinking agents such as glutaraldehyde [540,542,543,678,716,720] with the purpose to improve the mechanical properties of these biocomposites. Unfortunately, a further progress in this direction is restricted by a high cost, difficulty to control cross-infection, a poor definition of commercial sources of collagens, as well as by a lack of an appropriate technology to fabricate bone-resembling microstructures. Further details on CaPO_4_/collagen biocomposites might be found elsewhere [652,663,727].

### 4.5. Formulations with Other Bioorganic Compounds

The biggest practical problems with collagen type I are its cost and the poor definition of commercial sources of this material, which makes it difficult to follow up on well controlled processing. Therefore, collagen type I can be replaced by other compounds. One should notice, that, besides collagen, both human and mammalian bodies contain dozens types of various bioorganic compounds, proteins and biological macromolecules. Therefore, the substantial amount of them potentially might be used to prepare biocomposites with CaPO_4_. For example, a biologically strong adhesion (to prevent invasion of bacteria) between teeth and the surrounding epithelial tissues is attributed to a cell-adhesive protein, laminin [728]. In order to mimic the nature, a laminin/apatite biocomposite layer was successfully created on the surface of both titanium [729] and EVOH [730,731] using the biomimetic approach. A more complicated laminin/DNA/apatite biocomposite layer was found to be an efficient gene transfer system [732]. Further details on this subject are available in a topical review [733].

CaPO_4_/gelatin biocomposites are widely investigated as potential bone replacement biomaterials [253,383,384,385,386,387,388,389,390,409,446,447,448,559,560,561,562,563,564,734,735,736,737,738,739]. For example, gelatin foams were successfully mechanically reinforced by HA and then crosslinked by a carbodiimide derivative [253]. Such foams were shown to be a good carrier for antibiotic tetracycline [735]. Several biocomposites of CaPO_4_ with alginates have been prepared [387,556,557,560,655,740,741,742,743,744]. For example, porous HA/alginate composites based on hydrogels were prepared both biomimetically [655] and by using a freeze-drying technique [740]. More complicated formulations have been developed as well [745].

Various biocomposites of CaPO_4_ with chitosan [234,403,425,436,453,462,499,581,582,583,584,585,586,587,588,589,590,591,618,638,643,746,747,748,749,750,751,752,753,754,755,756,757] and chitin [392,452,758,759,760,761,762] are also very popular. For example, a solution-based method was developed to combine HA powders with chitin, in which the ceramic particles were uniformly dispersed [758,759]. Unfortunately, it was difficult to obtain the uniform dispersions. The mechanical properties of the final biocomposites were not very good; due to a poor adhesion between the filler and the matrix both the tensile strength and modulus were found to decrease with increase of the HA amount. Microscopic examination revealed that HA particles were intervened between the polymer chains, weakening their interactions and decreasing the entire strength [758,759]. Other manufacturing techniques might be found in the aforementioned references, for example, a HA/chitosan biocomposite was produced by a hydrothermal process from natural CaCO_3_/chitosan biocomposite of crab shells [751]. Similarly, HA/chitosan biocomposite was produced by a hydrothermal process from previously prepared DCPD/chitosan biocomposite [752]. Biocomposites of natural HA with chitosan were found to possess both a good hard tissue biocompatibility and an excellent osteoconductivity, which is suitable for artificial bone implants and frame materials of tissue engineering [748]. Data are available that addition of CaPO_4_ into chitosan improved cell attachment, provided a higher cell proliferation and well-spread morphology when compared to chitosan alone [584,754]. More complex formulations, such as silk fibers reinforced HA/chitosan [763] and HA/collagen/chitosan [764] biocomposites, have been studied as well. Besides biomedical applications, biocomposites of nano-sized HA with chitin/chitosan might be used for removal of Fe(III) [765] and fluorides [766,767] from aqueous solutions.

Biocomposites of CDHA with water-soluble proteins, such as BSA, might be prepared by a precipitation method [493,768,769,770,771]. In such biocomposites, BSA is not strongly fixed to solid CDHA, which is useful for a sustained release. However, this is not the case if a water/oil/water interfacial reaction route has been used [247]. To extend this subject, inclusion of DNA into CDHA/BSA biocomposites was claimed [247,772,773,774]. Furthermore, biocomposites of an unspecified CaPO_4_ with DNA [775,776], as well as those of nano-sized crystals of biomimetic apatite with C_60_ fullerene and Au-DNA nano-sized particles [777] were prepared as well.

Akashi and co-workers developed a procedure to prepare CaPO_4_-based biocomposites by soaking hydrogels in supersaturated by Ca^2+^ and PO_4_^3−^ ions solutions in order to precipitate CDHA in the hydrogels (up to 70% by weight of CDHA could be added to these biocomposites) [778]. This procedure was applied to chitosan; the 3D shape of the resulting biocomposite was controlled by the shape of the starting chitosan hydrogel [779]. Another research group developed biocomposites based on *in situ* CaPO_4_ mineralization of self-assembled supramolecular hydrogels [780]. Other experimental approaches are also possible [781].

**Figure 6:**
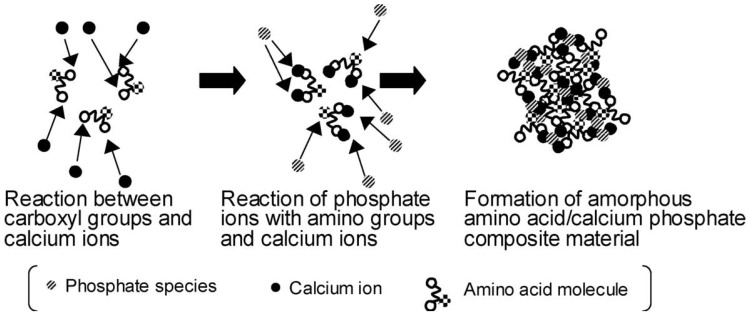
A proposed mechanism for the formation of amorphous calcium phosphates (ACP)/amino acid biocomposites in aqueous solutions. Reprinted from Ref. [786] with permission.

Various biocomposites of CDHA with glutamic and aspartic amino acids, as well as poly-glutamic and poly-aspartic amino acids have been prepared and investigated [277,278,782,783,784,785]. These (poly)amino acids were quantitatively incorporated into CDHA crystals, provoking a reduction of the coherent length of the crystalline domains and decreasing the crystal sizes. The relative amounts of the (poly)amino acid content in the solid phase, determined through HPLC analysis, increased with their concentration in solution up to a maximum of about 7.8 wt % for CDHA/aspartic acid and 4.3 wt % for CDHA/glutamic acid biocomposites. The small crystal dimensions, which implied a great surface area, and the presence of (poly)amino acids were suggested to be relevant for possible application of these biocomposites for hard tissues replacement [277,278,782,783,784,785]. A schematic description of a biocomposite formation from amino acids and ACP is shown in Figure 6 [786].

Furthermore, BCP (HA + β-TCP)/agarose macroporous scaffolds with controlled and complete interconnection, high porosity, thoroughly open pores and tailored pore size were prepared for tissue engineering application [787,788]. Agarose, a biodegradable polymer, was selected as the organic matrix because it was a biocompatible hydrogel, which acted as gelling agent leading to strong gels and fast room temperature polymerization. Porous scaffolds with the designed architecture were manufactured by combining a low temperature shaping method with stereo-lithography and two drying techniques. The biocompatibility of this BCP/agarose system was tested with mouse L929 fibroblast and human SAOS-2 osteoblast during different colonization times [789].

Fibrin sealants are non-cytotoxic, fully resorbable, biological matrices that simulate the last stages of a natural coagulation cascade, forming a structured fibrin clot similar to a physiological clot [790]. Biocomposites of CaPO_4_ with fibrin sealants might develop the clinical applications of bone substitutes. The 3D mesh of fibrin sealant interpenetrates the macro- and micro-porous structure of CaPO_4_ ceramics. The physical, chemical and biological properties of CaPO_4_ bioceramics and the fibrin glue might be cumulated in biocomposites, suitable for preparation of advanced bone grafts [791,792,793,794,795,796,797].

Furthermore, there are biocomposites of CaPO_4_ with bisphosphonates [798], silk fibroin [245,619,620,799,800,801,802,803,804], cellulose [805], chitosan + silk fibroin [806], chitosan derivatives [807], fibronectin [808], chondroitin sulfate [235,675,809], casein phosphopeptides [810], okra hydrocolloids [811], keratin hydrogel [812], amyloid fibrils [813], agarose [814] and vitamins [815]. Photopolymerisable formulations have been developed as well [816]. Besides, the reader’s attention is pointed out to an interesting approach to crystallize CDHA inside poly(allylamine)/poly(styrene sulfonate) polyelectrolyte capsules resulting in empty biocomposite spheres of micron size. Depending on the amount of precipitated CDHA, the thickness of the shell of biocomposite spheres can be varied between 25 and 150 nm. These biocomposite capsules might find application as medical agents for bone repairing and catalytic microreactors [817].

### 4.6. Injectable Bone Substitutes (IBS)

With the development of minimally invasive surgical methods, for example percutaneous surgery, directly injectable biomaterials are needed. The challenge is to place a biomaterial at the site of surgery by the least possible invasive method. In this regard, IBS appear to be a convenient alternative to solid bone-filling materials. They represent ready-to-use suspensions of CaPO_4_ microspheres [818,819], nano-sized rods [820] or powder(s) in a liquid carrier phase. However, addition of other phases, such as calcium sulfate [821], is possible. IBS look like opaque viscous pastes with the rheological properties, sufficient to inject them into bone defects by means of surgical syringes and needles. Besides, IBS could be easily produced in a sterile stage. Their stable composition and mechanical properties are suitable for reproducibility of the biological response [822,823]. All types of IBS are divided into two major groups: self-setting formulations (see Section 4.2.) and those, which do not set. The latter ones are described here.

IBS requires suitable rheological properties to ensure bonding and cohesion of the mineral phase *in situ* with good cell permeability. Usually, the necessary level of viscosity is created by addition of suitable water-soluble polymers [92,251,564,824,825,826,827,828,829,830,831,832]. However, IBS based on hydrogels are known as well [833,834,835]. Therefore, the majority of CaPO_4_-containing IBS formulations might be considered as a subgroup of CaPO_4_/polymer biocomposites. For example, an IBS was described that involved a silanized hydroxyethylcellulose carrier with BCP (HA + β-TCP). The suspension is liquid at pH within 10–12, but gels quickly at pH < 9 [826]. Injectable composites can be formed with β-TCP to improve mechanical integrity [480]. Similarly, a polydioxanone*-co-*glycolide-based biocomposite reinforced with HA or β-TCP can be used as an injectable or moldable putty [827]. During the crosslinking reaction following injection, carbon dioxide is released allowing the formation of interconnected pores. Furthermore, HA/poly(L-lactide*-co-*ε-caprolactone) biocomposite microparticles were fabricated as an injectable scaffold via the Pickering emulsion route in the absence of any molecular surfactants. A stable injectable oil-in-water emulsion was obtained using water dispersed HA nano-sized crystals as the particulate emulsifier and a dichloromethane solution of poly(L-lactide*-co-*ε-caprolactone) as an oil phase [251]. CaPO_4_-containing IBS based on other (bio)organic hydrogels, such as gelatin [564], CMC [830], oligo(poly(ethylene glycol) fumarate) [828,829], chitosan + collagen [831] have been developed as well. In addition, photocrosslinkable formulations are known [832].

Daculsi *et al.* developed viscous IBS biocomposites based on BCP (60% HA + 40% β-TCP) and 2% aqueous solution of HPMC that was said to be perfectly biocompatible, resorbable and easily fitted bone defects (due to an initial plasticity) [825,836,837,838,839,840,841,842,843]. The best ratio BCP/HPMC aqueous solution was found to be at ~65/35 w/w. To extend this subject further, IBS might be loaded by cells [844,845], radiopaque elements [846] or microparticles [847], as well as functionalized by nucleic acids [848]. Self-hardening formulations, based on Si-HPMC hydrogel, are known as well [844]. The list of the commercially available CaPO_4_-based IBS formulations is presented in Table 5 [848].

**Table 5:** A list of some commercial non-setting CaPO_4_-based injectable bone substitutes (IBS) and pastes with indication of producer, product name, composition (when available) and form [848].

Producer	Product name	Composition	Form
ApaTech (UK)	Actifuse™	HA, polymer and aqueous solution	pre-mixed
	Actifuse™ Shape; Actifuse™ ABX	Si-substituted CaPO_4_ and a polymer	pre-mixed
Baxter (US)	TricOs Τ; TricOs	BCP (60% HA, 40% β-TCP) granules and Tissucol (fibrin glue)	to be mixed
Berkeley Advanced Biomaterials	Bi-Ostetic Putty	not disclosed	not disclosed
BioForm (US)	Calcium hydroxylapatite implant	HA powder embedded in a mixture of glycerine, water and CMC	pre-mixed
Biomatlante (FR)	In’Oss™	BCP granules (60% HA, 40% β-TCP; 0.08–0.2 mm) and 2% HPMC	pre-mixed
	MBCP Gel^®^	BCP granules (60% HA, 40% β-TCP; 0.08–0.2 mm) and 2% HPMC	pre-mixed
	Hydr’Os	BCP granules (60% HA, 40% β-TCP; micro- and nano-sized particles) and saline solution	pre-mixed
Degradable solutions (CH)	Easy graft™	β-TCP or BCP granules (0.45–l.0 mm) coated with 10 μm PLGA, N-methyl-2-pyrrolydone	to be mixed
Dentsply (US)	Pepgen P-15^®^ flow	HA (0.25–0.42 mm), P-15 peptide and aqueous Na hyaluronate solution	to be mixed
DePuy Spine (US)	Healos^®^ Fx	HA (20%–30%) and collagen	to be mixed
Fluidinova (P)	nanoXIM TCP	β-TCP (5% or 15%) and water	pre-mixed
	nanoXIM HA	HA (5%, 15%, 30% or 40%) and water	pre-mixed
Integra LifeSciences (US)	Mozaik Osteoconductive Scaffold	β-TCP (80%) and type 1 collagen (20%)	to be mixed
Mathys Ltd (CH)	Ceros^®^ Putty/cyclOS^®^ Putty	β-TCP granules (0.125–0.71 mm; 94%) and recombinant Na hyaluronate powder (6%)	to be mixed
Medtronic (US)	Mastergraft^®^	BCP (85% HA, 15% β-TCP) and bovine collagen	to be mixed
Merz Aesthetics (GER)	RADIESSE^®^	HA particles suspended in a gel	pre-mixed
Osartis / ΑΑΡ (GER)	Ostim^®^	Nanocrystalline HA (35%) and water (65%)	pre-mixed
Smith & Nephew (US)	JAXTCP	β-TCP granules and an aqueous solution of 1.75% CMC and 10% glycerol	to be mixed
Stryker (US)	Calstrux™	β-TCP granules and CMC	to be mixed
Teknimed (FR)	Nanogel	HA (100 – 200 nm) (30%) and water (70%)	pre-mixed
Therics (US)	Therigraft™ Putty	β-TCP granules and polymer	pre-mixed
Zimmer (US)	Collagraft	BCP granules (65% HA, 35% β-TCP; 0.5–1.0 mm), bovine collagen and bone marrow aspirate	to be mixed

The advanced characteristics of IBS come from their good rheological properties and biocompatibility and the ease of tissue regeneration. Although the fabrication of IBS biocomposites in most cases improved the mechanical properties of the system and provided the material with resistance to fluids penetration, these achievements were limited by the amount of polymer that can be added to the paste. For instance, Mickiewicz *et al.* reported that after a critical concentration (that depended on the type and molecular weight of the polymer, but was always around 10%), the polymer started forming a thick coating on the crystal clusters, preventing them from interlocking, originating plastic flow and, as a consequence, decreasing mechanical properties [493]. More to the point, Fujishiro *et al.* reported a decrease in mechanical properties with higher amounts of gel, which was attributed to formation of pores due to leaching of gelatin in solution [486]. Therefore, it seems that mechanical properties, although improved by the addition of polymers, are still a limitation for the application of CaPO_4_-based IBS formulations in load-bearing sites [125]. Further details on IBS are available elsewhere [822,823].

### 4.7. Biocomposites with Inorganic Compounds, Carbon and Metals

To overcome the problem of poor mechanical properties of CaPO_4_ bioceramics, suitable biocomposites of CaPO_4_ reinforced by various inorganic materials, glasses and metals have been developed as well. Such biocomposites are mainly prepared by the common ceramic processing techniques such as thermal treatment after kneading [849,850,851], powder slurry coating [852] and metal-sol mixing [853]. For example, HA was combined with Bioglass^®^ (Novabone Products, Alachua, FL) [854,855] and with other glasses [856] to form glass-ceramics biocomposites. Other reinforcement materials for CaPO_4_ could be differentiated by either shape of the fillers, such as particles [857,858], platelets [859,860], whiskers [520,861,862,863], fibers [864,865,866,867,868], or their chemical composition. Namely, zirconia and PSZ [849,850,851,852,861,869,870,871,872,873,874,875,876,877,878,879,880,881], alumina [857,860,882,883,884,885,886,887], titania [888,889,890,891,892,893,894,895,896] and their mixtures [897], other oxides and their mixtures [863,898,899,900,901,902,903,904,905], silica and/or glasses [906,907,908,909,910,911,912,913,914,915], wollastonite [916,917,918,919,920], mullite [921,922,923,924], various metals and alloys [495,610,611,866,925,926,927,928,929,930,931,932,933,934,935,936,937,938,939,940,941,942,943], calcium sulfate [944,945,946,947,948,949], calcium carbonate [950,951], silicon carbide [623,862], barium titanate [952], zeolite [953], boron nitride [954], zirconium nitride [955], carbon [956] and several other materials [957,958,959] were added to CaPO_4_ to improve reliability. In addition, CaPO_4_-based biocomposites possessing piezoelectric properties have been proposed [960]. Interestingly, Fe_3_O_4_/HA composites possess photocatalytic properties [902,903]. More complicated formulations, such as HA/aluminum oxide/carbon nanotubes [961], have been developed as well. Unfortunately, significant amounts of the reinforcing phases are needed to achieve the desired properties and, as these materials are either bioinert, significantly less bioactive than CaPO_4_ or not bioresorbable, the ability of the biocomposites to form a stable interface with bone is poorer if compared with CaPO_4_ bioceramics alone. Due to the presence of bioinert compounds, such formulations occasionally are called bioinert/bioactive composites [908]. The ideal reinforcement material would impart mechanical integrity to a biocomposite at low loadings, without diminishing its bioactivity.

There are several types of HA/glass biocomposites. The first one is also called bioactive glass-ceramics. A dense and homogeneous biocomposite was obtained after a heat treatment of the parent glass, which comprised ~38 wt % oxy-FAP (Ca_10_(PO_4_)_6_(O,F)_2_) and ~34 wt % β-wollastonite (CaO·SiO_2_) crystals, 50–100 nm in size in a MgO-CaO-SiO_2_ glassy matrix [916,917,918,919,920]. A-W glass-ceramics is an assembly of small apatite particles effectively reinforced by wollastonite. The bending strength, fracture toughness and Young’s modulus of A-W glass-ceramics are the highest among bioactive glass and glass-ceramics, enabling it to be used in some major compression load bearing applications, such as vertebral prostheses and iliac crest replacement. It combines a high bioactivity with the suitable mechanical properties [962]. β-TCP/wollastonite biocomposites are also known [963,964,965]. More complicated formulations have been developed as well. For example, A-W/HDPE composite (AWPEX) biomaterials have been designed to match the mechanical strength of human cortical bone and to provide favorable bioactivity, with potential use in many orthopedic applications [966,967,968,969]. Similar can be said on A-W/silk fibroin biocomposites [970]. Other examples comprise wollastonite-reinforced HA/Ca polycarboxylate [971], glass-reinforced HA/polyacrylate [972], as well as collagen- [973] and gelatin- [974] calcium phosphate silicate/wollastonite biocomposites.

HA/glass biocomposites can be prepared by simple sintering of appropriate HA/glass powder mixtures [975,976,977,978]. If sintering is carried out below 1000 °C, HA does not react with the bioactive glass [976,977] or this reaction is limited [978]. Besides, reaction between HA and glasses depends on the glass composition. In another approach, small quantities of bioactive glass have been added to HA bioceramics in order to improve densification and/or mechanical properties [23]. In addition, biocomposites might be sintered from HA and silica [908]. In general, bioactive glass-ceramics maintain a high strength for a longer time than HA bioceramics under both the *in vitro* and *in vivo* conditions [910].

Due to a huge difference in shapes, it is a challenge to prepare homogeneous mixtures of CaPO_4_ and carbon nanotubes: “one can imagine something similar to achieving a homogeneous mixture of peas and spaghetti” [979] (p. 7). Nevertheless, different strategies might be employed to prepare CaPO_4_/carbon nanotube biocomposites. For example, apatites might be chemically synthesized by using carboxyl functionalized carbon nanotubes as a matrix [238,239,240,241,980,981,982,983,984]. Physico-chemical characterization of these biocomposites showed that nucleation of CDHA initiates through the carboxyl group [238]. Hot pressing [985], compaction [986], plasma spraying [987], laser surface alloying [988,989,990], spark plasma sintering [991], shear mixing [992], sol-gel [993] and precipitation [994] techniques might be applied as well. Due to carbon oxidation at elevated temperature, sintering of CaPO_4_/carbon nanotube biocomposites must be performed in a deoxidizing atmosphere [995]. The research on CaPO_4_ (up to now, only apatite)/carbon nanotube biocomposites is in its early stages, with the first papers published in 2004 [980]. Due to this reason, the mechanical property data for such biocomposites have been reported only in few papers; however, these results are encouraging. For example, Chen *et al.*, performed nano-indentation tests on biocomposite coatings to give hardness and Young’s modulus values [990]. They found that the higher the loading of the nanotubes, the better the mechanical properties. Namely, at 20 wt % loading, hardness was increased by ~43% and Young’s modulus by ~21% over a single-phase HA coating [990]. Scratching test results indicated that as alloyed HA biocomposite coatings exhibited improved wear resistance and lower friction coefficient with increasing the amount of carbon nanotubes in the precursor material powders [989]. Additionally, measurements of the elastic modulus and hardness of the biocomposite coatings indicated that the mechanical properties were also affected by the amount of carbon nanotubes [988]. Another research group performed compression tests on bulk HA/carbon nanotubes biocomposites and found an increase in strength over single-phase HA [980]. However, the highest compressive strength they achieved for any material was only 102 MPa, which is similar to that of cortical bone but much lower than the typical values for dense HA [979]. More complex formulations, such as poly-L-lysine/HA/carbon nanotube hybrid biocomposites, have been also developed [996]. Furthermore, CaPO_4_/carbon nanotube biocomposites might be immobilized by hemoglobin [997]. Unfortunately, carbon nanotubes are very stable substances; they are neither bioresorbable nor biodegradable. Therefore, during *in vivo* bioresorption, the nanotubes will get into the human body from the biocomposite matrix and might cause uncertain health problems. Certainly, this problem must be solved. To conclude the carbon subject, one should mention on application of carbon fibers of microscopic dimensions [998,999,1000], nanodimensional diamonds [1001], graphene [1002,1003,1004,1005,1006,1007], fullerenes [777] and its derivatives [1008] to reinforce CaPO_4_ bioceramics.

As seen from the amount of the references, CaPO_4_/zirconia biocomposites appear to be a hot topic of the research. The main disadvantage of HA reinforced by PSZ is degradation of zirconia in wet environments [861,870,871,874]. Transformation of the tetragonal ZrO_2_ to the monoclinic phase on the surface results in formation of microcracks and consequently lowers the strength of the implant [1009,1010].

Various biocomposites of CaPO_4_ with metals and alloys have been fabricated as well [495,610,611,866,925,926,927,928,929,930,931,932,933,934,935,936,937,938,939,940,941,942,943]. For example, an HA-based biocomposite reinforced with 20 vol.% of Ti particles was fabricated by hot pressing [927]. Besides, biocomposites of CaPO_4_ with metals might be prepared by powder metallurgy processing [928,930,941,942] and mechanical compaction followed by sintering [611,936,939]. Furthermore, silver nanoparticles/HA biocomposites could be synthesized through γ-irradiation reduction of silver ions incorporated into HA particles [610]. At high temperatures, the presence of Ti metal phase was found to promote dehydration and decomposition of HA into β-TCP and TTCP [927,928,939,1011] and/or partial formation of β-TCP and calcium titanates instead of HA [603,930,942,1011]. Comparing with pure HA bioceramics manufactured under the same conditions, the HA/Ti biocomposites possessed a higher fracture toughness, bending strength, work of fracture, which is better suitable for the biomedical applications [936]. However, the mechanical properties appeared to be not high enough to use HA/Ti biocomposites in load-bearing applications. Luckily, the histological evaluations revealed that HA/Ti biocomposites could be partially integrated with newborn bone tissues after 3 weeks and fully osteointegrated at 12 weeks *in vivo* [927]. Similar findings had been earlier made for HA bioceramics reinforced by addition of silver particulates (5–30 vol.%) and subsequent sintering of the HA/Ag powder compacts [925,926]. Besides, addition of silver imparts an antimicrobial activity [932]. Other studies on CaPO_4_/Ti biocomposites are available elsewhere [929,931,942]. In all the aforementioned studies, CaPO_4_ was used as a matrix; however, the opposite situation is also possible. There is a study, in which composites made of Mg3Zn0.8Zr alloy as a matrix and β-TCP particles as reinforcements were prepared for investigating the effect of β-TCP spherical particles sized in 100 nm on the microstructure of that alloy [1012].

To conclude this part, biocomposites consisting of CaPO_4_ only should be briefly described. First, all types of biphasic, triphasic and multiphasic CaPO_4_ formulations should be mentioned [412]. For example, in 1980-s, BCP was called as “TCP ceramics complexed with HA” [1013]. Even nowadays BCP is occasionally called as a “nanocomposite” [1014]. Furthermore, fluoridated HA (described by a chemical formula Ca_10_(PO_4_)_6_(OH)_2−*x*_F*_x_*, where 0 < *x* < 2) might be mentioned as composites [1015]; however, an applicability of the “composite” term for such systems is doubtful. One should better consider 70% HA-powder + 30% HA-whisker biocomposites, which were fabricated by pressureless sintering, hot pressing and hot isostatic pressing. These biocomposites were found to exhibit an improved toughness, attaining the lower fracture-toughness limit of bone without a decrease of bioactivity and biocompatibility [1016,1017]. Besides, a dual HA biocomposite that combined two HA materials with different porosities: HA with 75% porosity, for bone ingrowth and HA with 0% porosity, for load bearing was manufactured. This dual HA biocomposite appeared to be suitable for use as an implant material for spinal interbody fusion as a substitute for iliac bone grafts, which could eliminate the disadvantages associated with autograft harvesting [1018]. A biodegradable biocomposite porous scaffold comprising a β-TCP matrix and nano-sized fibers of HA was developed and studied for load-bearing bone tissue engineering. The nano-sized fibers of HA were prepared by a biomimetic precipitation method, the inclusion of which significantly enhanced the mechanical property of the scaffold, attaining a compressive strength of 9.87 MPa, comparable to the high-end value (2–10 MPa) of cancellous bone [1019]. Furthermore, HA/β-TCP biocomposites with different β-TCP content (10, 20, and 30 wt %) were prepared by wet mixing of HA and β-TCP powders, compaction of the powder mixtures and sintering [1020]. Finally, it is interesting to mention on a successful reinforcement of carbonateapatite porous blocks by newly prepared carbonateapatite crystals (*i.e*., by the same compound; thus, a biocomposite of two different carbonateapatites was obtained) [1021]. First, a calcium salt was introduced to micropores of carbonateapatite blocks. Then, the calcium salt was carbonated to form calcite inside the micropores of the carbonateapatite blocks by exposing the blocks to carbon dioxide at the second step. On the third step, the blocks were immersed in a Na_2_HPO_4_ aqueous solution. In this process, calcite inside the micropores of the carbonateapatite blocks was transformed to carbonateapatite and the newly formed crystals of carbonateapatite entangled on those of the existing carbonateapatite blocks. Due to bonding between the newly formed carbonateapatite crystals and the existing ones in the carbonateapatite blocks, a mechanical strength of the blocks became ~1.5 times higher when compared to that before the treatment [1021].

### 4.8. Functionally Graded Formulations

Although, in most cases, the homogeneous distribution of filler(s) inside a matrix is required [347], there are composites, in which this is not the case. For example, functionally graded materials (FGM) might be characterized by the intentional variations in composition and/or structure gradually over volume, resulting in corresponding changes in the properties of the composite. The main feature of such materials is the almost continuously graded composition that results in two different properties at the two ends of the structure. Such composites can be designed for specific function and applications. Various approaches based on the bulk (particulate processing), pre-form processing, layer processing and melt processing are used to fabricate the functionally graded materials.

Bone is a biologically formed composite with variable density ranging from very dense and stiff (cortical bone) to a soft and foamed structure (trabecular bone). Normally the outer part of long bones consists of cortical bone with the density decreasing towards the core, where the trabecular bone is found. The trabecular bone is porous and the porosity is filled with osseous medulla [14,15]. This brief description clearly indicates that bones are natural functionally graded composites.

**Figure 7:**
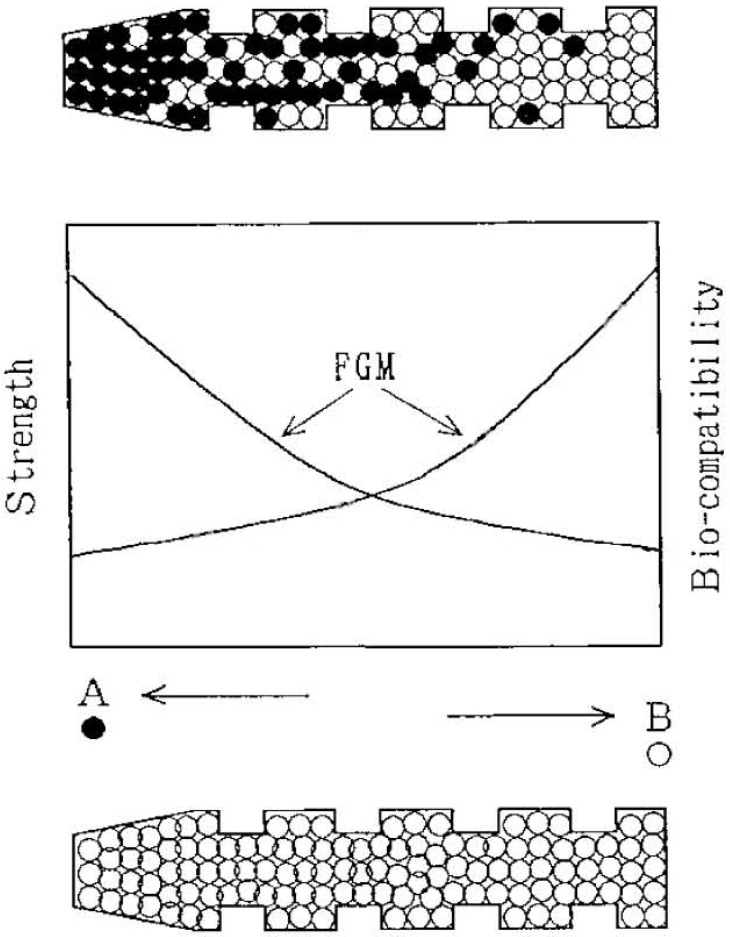
Expected properties of functionally graded biocomposite dental implant. For comparison, the upper drawing shows a functionally graded implant and the lower one shows a conventional uniform implant. The properties are shown in the middle. The implant with the composition changed from a biocompatible metal (Ti) at one end (left in the figure), increasing the concentration of bioceramics (hydroxyapatite (HA)) toward 100% HA at the other end (right in the figure), could control both mechanical properties and biocompatibility without an abrupt change due to the formation of discrete boundary. This functionally gradient material (FGM) biocomposite was designed to provide more titanium for the upper part where occlusal force is directly applied and more HA for the lower part, which is implanted inside the jawbone. Reprinted from Ref. [1027] with permission.

The concept of FGM has been increasingly used for biomaterial design and currently it remains to be an important area of the research. For example, many studies have been performed to fabricate porosity-graded CaPO_4_ bioceramics in attempts to mimic the porous structure of bones [1022,1023,1024,1025]. This is a structural approach to fabricate FGM. Besides, there is a compositional approach. For example, functionally graded composite skull implants consisting of polylactides, carbonateapatite and CaCO_3_ have been prepared [312,313]. In addition, powder metallurgy methods have been used to fabricate HA/Ti functionally graded biocomposite dental implants offering the biocompatible HA on the tissue side and titanium on the outer side for mechanical strength [1026,1027,1028]. The graded structure in the longitudinal direction contains more Ti in the upper section and more HA in the lower section. Actually, in the upper section the occlusal force is directly applied and Ti offers the required mechanical performance; in the lower part, which is implanted inside the bone, the HA confers the bioactive and osteoconductive properties to the material [1026]. Since the optimum conditions of sintering for Ti and HA are very different, HA/Ti functionally graded biocomposites are difficult to fabricate and the sintering conditions for their mixtures are obliged to compromise. The expected properties of this implant are shown in Figure 7 [1027]. Such biocomposites might be both symmetrical [1029] and asymmetrical [1030].

A series of functionally graded CaPO_4_ coatings incorporated with various percentages of silver were deposited on titanium substrates using ion beam-assisted deposition. The analysis of the coating’s cross-section revealed a decreased crystallinity as well as a distribution of nano-sized (10–50 nm) silver particles from the coating/substrate interface to top surface [1031]. Similarly, compositionally graded HA/Ti biocomposite coatings were prepared by rf-plasma spraying [1032]. In addition, both laser cladding [1033] and combinatorial matrix-assisted pulsed laser evaporation [1034] techniques were used to put down gradient deposits of carbon nanotubes/HA and Sr-substituted HA and zoledronate-modified HA, respectively. A functionally graded HA/PMMA biocomposite was developed based on sedimentary HA distributions in a PMMA viscous fluid, using a centrifuge to avoid stress convergence on the interface. The stress-strain curves of this biocomposite showed a sufficient strength for biomedical applications along with the relaxation of brittleness and fragility [473]. A compositionally graded collagen/nanodimensional HA biocomposite scaffold might be prepared by an *in situ* diffusion method. Chemical and microstructural analysis revealed a gradient of the Ca to P ratio across the width of the scaffold template, resulting in the formation of a Ca-rich side and a Ca-depleted side of scaffold. The Ca-rich side featured low porosity and agglomerates of the nanodimensional HA crystallites, while the Ca-depleted side featured higher porosity and nanodimensional HA crystallites integrated with collagen fibrils to form a porous network structure [1035]. A three-layered graded biocomposite membrane, with one face of 8% nanodimensional carbonateapatite/collagen/PLGA porous membrane, the opposite face of pure PLGA non-porous membrane and the middle layer of 4% nanodimensional carbonateapatite/collagen/PLGA as the transition was prepared through the layer-by-layer casting method [552]. A similar PLGA/nano-HA/lauric acid graded biocomposite was prepared as well [1036]. Functionally graded non-woven meshes of PCL incorporated by nano-sized particles of β-TCP were prepared using a hybrid twin-screw extrusion/electrospinning process [1037]. A functionally graded HA/silk fibroin biocomposite was prepared by pulse electric current sintering [1038]. HA/glass FGM layers were coated on titanium alloy (Ti6Al4V) substrates. The design of these layers and the use of the glass were for achieving a strong bonding between the FGM layered coatings and the substrates [1039,1040].

**Figure 8:**
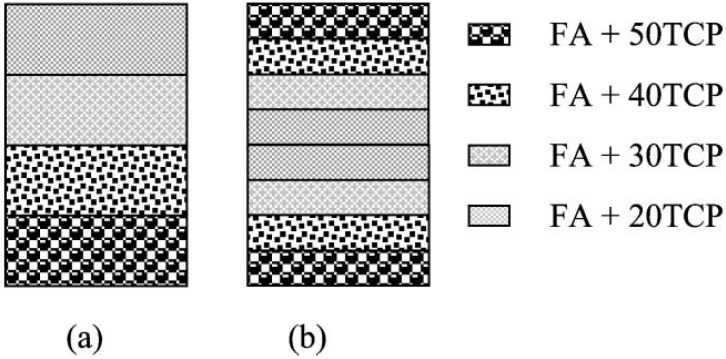
A schematic diagram showing the arrangement of the FA/β-TCP biocomposite layers. (**a**) A non-symmetric functionally gradient material (FGM); (**b**) symmetric FGM. Reprinted from Ref. [1041] with permission.

Functionally graded β-TCP/FA biocomposites combine the biostability of FA with the bioresorbable properties of β-TCP. An interesting multilayered (each layer of 1 mm thick) structure consisting of β-TCP/FA biocomposites with different molar ratios has been prepared, giving rise to formation of a FGM (Figure 8). After implantation, the preferential dissolution of β-TCP phase would result in functionally gradient porosity for bone ingrowth [1041]. In addition, compositionally graded HA-alumina-zirconia biocomposites were prepared by the same multilayered technique [1042]. Functionally graded fluoridated HA with a gradient of fluoride [1043] and carbonated HA with a gradient of carbonate [1044] were synthesized as well. HA/zirconia graded biocomposites were fabricated to enhance the mechanical properties of HA while retaining its bone bonding property [879]. TiO_2_ and HA were found to be a good combination for FGM providing both a gradient of bioactivity and a good mechanical strength [1045]. Besides, graded HA/CaCO_3_ biocomposite structures for bone ingrowth were developed [1046]. Functionally graded composite skull implants consisting of polylactides, carbonateapatite and CaCO_3_ are known as well [312,313]. Thus, the research in this field is quite promising but currently the mechanical properties of the available biocomposites do not match the similar properties of bones [1047].

### 4.9. Biosensors

A biosensor is a device for detection of an analyte that combines a biological component with a physicochemical detector component. Very briefly, it consists of three parts: a sensitive biological element; a transducer or a detector element that transforms the signal resulting from the interaction of the analyte with the biological element into another signal; and associated electronics that is primarily responsible for the display of the results in a user-friendly way [1048].

The surface of biologically relevant CaPO_4_ (CDHA, HA, α-TCP, β-TCP, DCPD, DCPA) has an excellent ability of adsorption for functional biomolecules such as proteins, albumins, DNA, as well as some other types of chemicals. Therefore, several CaPO_4_-based biocomposites and hybrid biomaterials were found to be applicable for manufacturing of biosensors [996,1049,1050,1051,1052,1053,1054,1055,1056]. For example, formation of poly-L-lysine/HA/carbon nanotube hybrid nanodimensional particles was described and a general design strategy for an immunosensing platform was proposed based on adsorption of antibodies onto this biocomposite [996]. In another paper, a hybrid material formed by assembling of nanodimensional particles of gold onto nano-sized HA was employed for the interface design of piezoelectric immunosensor, on which the antibodies were bound. The developed sensing interface appeared to possess some advantages, such as activation-free immobilization and high antigen-binding activities of antibodies, over using nano-sized either HA or gold alone [1050]. A novel tyrosinase biosensor based on nano-sized HA/chitosan composite has been developed for the detection of phenolic compounds [1054]. Further details on the subject are available in the aforementioned references.

Up to date, not many papers have been published on the biosensor application of CaPO_4_-based biocomposites and hybrid biomaterials. Presumably, this subject will be further developed in the future and, perhaps, sometime implantable biosensors will be designed to perform the continuous concentration monitoring of the important biological macromolecules *in vivo*. Possibly, those implantable biosensors will be able to use an electric power, generated by DCPD/polymer composite-based battery devices [432,433].

## 5. Interactions among the Phases

An important aspect that should be addressed in details is a mutual interaction between CaPO_4_ and other phases in biocomposites and hybrid biomaterials. In general, an interaction among the phases in any composite can be either mechanical, when it results from radial compression forces exerted by the matrix on the filler particles (for example, developed during cooling due to thermal contraction), or chemical, when the reactivity of the filler towards the matrix has an important role. In the latter case, it is important to distinguish a physical interaction from chemical bonding [219]. According to Wypych [1057], physical interaction is more or less temporary, implicating hydrogen bonding or van der Waals forces, whereas chemical bonding is stronger and more permanent, involving covalent bond formation. Thus, a chemical interfacial bond among the phases is preferred to achieve a higher strength of a composite. The magnitude of the interfacial bond among the phases determines how well a weak matrix transmits stress to the strong fibers. However, while a bond among the matrix and reinforcements must exist for the purpose of stress transfer, it should not be so strong that it prevents toughening mechanisms, such as de-bonding and fiber pullout [979].

There is still doubt as to the exact bonding mechanism among bone minerals (biological apatite) and bioorganics (collagen), which undoubtedly plays a critical role in determining the mechanical properties of bones. Namely, bone minerals are not directly bonded to collagen but through non-collagenous proteins that make up ~3% of bones (Table 1) and provide with active sites for biomineralization and for cellular attachment [22]. In bones, the interfacial bonding forces are mainly ionic bonds, hydrogen bonds and hydrophobic interactions, which give the bones the unique composite behavior [322]. There is an opinion that, opposite to bones, there is no sign of chemical bonding among the phases in conventional CaPO_4_/collagen biocomposites, probably due to a lack of suitable interfacial bonding during mixing [703]. However, this is not the case for phosphorylated collagens [690]. Readers skilled in computer modeling are forwarded to simulations of the interactions between collagen peptides and HA surfaces [1058] as well as between three polymers (PE, PA and PLA) and HA surfaces [1059], respectively.

**Figure 9:**
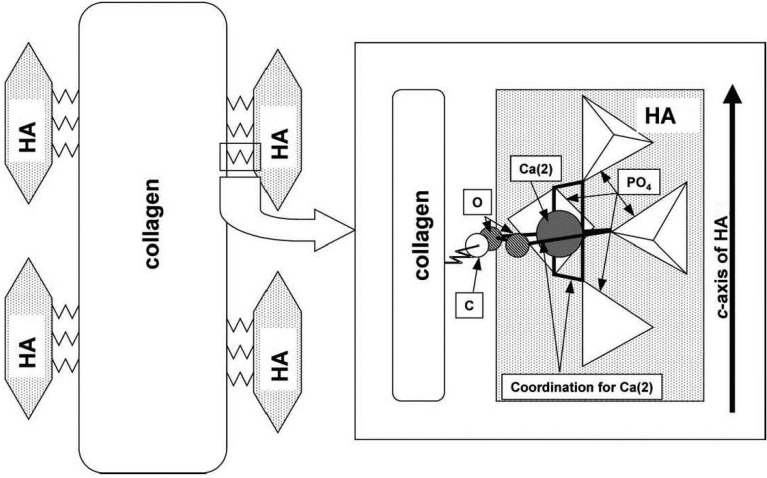
A schematic drawing of the relation between self-organization (directional deposition of HA on collagen) and interfacial interaction in biocomposites. Direction of interaction between HA and collagen is restricted by covalent bond between COO and Ca(2) to maintain regular coordination number of 7. Reprinted from Ref. [688] with permission.

To study possible interactions among the phases, Fourier-transformed infrared (FTIR) spectra of some CaPO_4_-based biocomposites and collagen films were collected and transformed into absorption spectra using the Kramers-Kronig equation to demonstrate energy shifts of residues on the apatite/collagen interface. After comparing FTIR spectra of biocomposites and collagen films in detail, red shifts of the absorption bands for C–O bonds were observed in the spectra of the biocomposites. These red shifts were described as a decrease of bonding energies of C–O bonds and assumed to be caused by an interaction to Ca^2+^ ions located on the surfaces of apatite nano-sized crystals, as shown in Figure 9 [688]. Another proof of a chemical interaction between apatite and collagen was also evaluated in FTIR spectra of CDHA/collagen biocomposites, in which a shift of the band corresponding to –COO^−^ stretching from 1340 to 1337 cm^−1^ was observed [654,655]. More to the point, nucleation of apatite crystals onto collagen through a chemical interaction with carboxylate groups of collagen macromolecules has been reported [1060,1061,1062].

**Figure 10:**
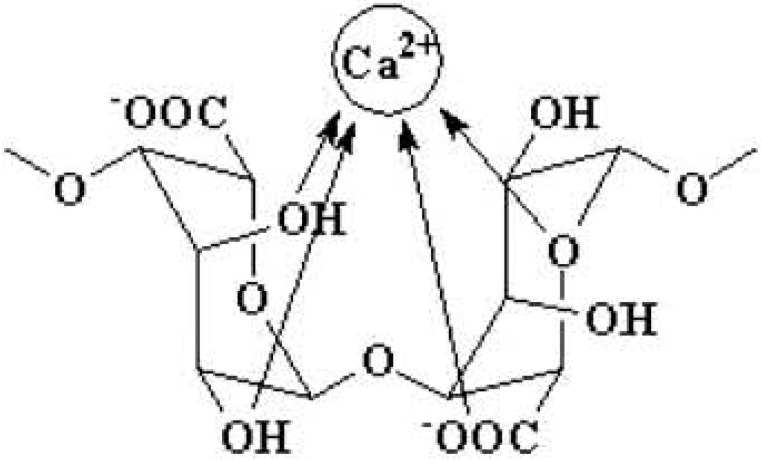
A schematic diagram of Ca^2+^ ion binding with alginate chains. Reprinted from Ref. [655] with permission.

FTIR spectroscopy seems to be the major tool to study a possible chemical bonding among the phases in CaPO_4_-based biocomposites and hybrid biomaterials [212,243,276,283,284,379,437,542,555,568,571,573,577,580,591,612,617,620,628,655,690,737,738,777,806,835,1063,1064,1065,1066,1067,1068,1069]. For example, the characteristic bands at 2918, 2850 and 1472 cm^−1^ for the hydrocarbon backbone of PE appeared to have zero shift in an HA/PE biocomposite. However, in the case of PA, several FTIR bands indicated that the polar groups shifted apparently: the bands at 3304, 1273 and 692 cm^−1^ derived from stretching of N–H, stretching of C–N–H and vibrating of N–H moved to 3306, 1275 and 690 cm^−1^, respectively, in the HA/PA biocomposites. Furthermore, both stretching (3568 cm^−1^) and vibrating (692 cm^−1^) modes of hydroxide in HA moved to 3570 and 690 cm^−1^ in the HA/PA biocomposites, respectively, indicating the formation of hydrogen bonds. Besides, bands at 1094 and 1031 cm^−1^ of PO_4_ modes also shifted to 1093 and 1033 cm^−1^ in HA/PA biocomposites. The bands shift in a fingerprint area indicated that the hydroxide and orthophosphate on the surface of HA might interact with plentiful carboxyl and amino groups of PA through nucleophilic addition [212]. Comparable conclusions were made for HA/PVA [577], CDHA/alginate [655], ACP/PPF [437], HA/maleic anhydride [284], HA/carboxylated PU [1069] and β-TCP/PLLA [379] biocomposites, in which weak chemical bonds were considered to form between Ca^2+^ ions located on the HA, CDHA, ACP or β-TCP surface, respectively, and slightly polarized O atoms of C=O bonds in the surrounding bioorganic compounds. The data obtained suggested that crystallization of CaPO_4_ in chitosan-containing solutions was substantially modulated by a chemical interaction of the components; apparently, a part of calcium was captured by chitosan and did not participate in the formation of the main mineral phase [1068]. Schematically, this type of the chemical interaction is shown in Figure 10 [655].

Except of FTIR spectroscopy, other measurement techniques are also able to show some evidences of a chemical interaction among the phases in CaPO_4_-based biocomposites and hybrid biomaterials [276,379,571,573,577,1064,1065,1066,1067,1068,1069,1070,1071]. For example, for nano-sized crystals of CDHA/alendronate such evidences were observed by thermogravimetric analysis: DTG plots of the crystals appeared to be quite different from those obtained from mechanical mixtures of CDHA and calcium alendronate with similar compositions [1070]. Analogous DTG results were obtained for nano-sized HA/PVA biocomposites [577]. In the case of biocomposites of nano-sized HA with PA, a hydrogen bonding among the phases was detected by differential scanning calorimetry technique [571]. Similar results were obtained in another study [1065]. One more example comprises application of the dynamic mechanical analysis to investigate softening mechanism of β-TCP/PLLA biocomposites [379]. As to biocomposites of nano-sized HA with PVAP, some indirect evidences of a chemical bonding among the phases were found by X-ray diffraction and thermogravimetric analysis [276]. A strong structural correlation between the orientation of FA crystallites and gelatin within the FA/gelatin composite spheres was discovered that indicated to a substantial reorganization of the macromolecular matrix within the area of a growing aggregate [366]. In addition, chemical interactions between HA and organic molecules have been elucidated using *ab initio* calculation methods [1072].

**Figure 11:**
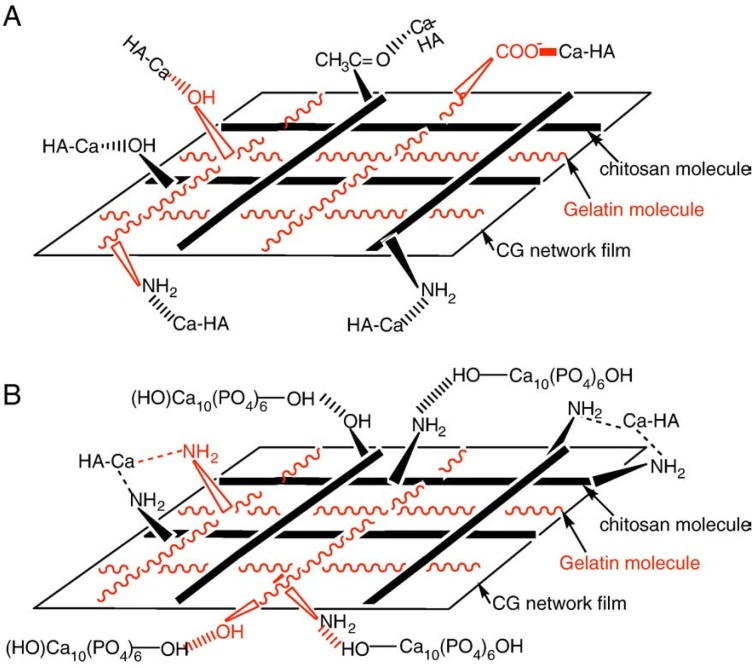
Possible interactions between a chitosan-gelatin (CG) network and HA crystals in HA/CG biocomposites: (**A**) In the case of a nano-dimensional HA (nHA); (**B**) In the case of a micro-dimensional HA (mHA). According to the authors: “When nHA formed on the surface of CG network via biomineralization, the corresponding ion interaction is the main drive force. However, as the mHA crystals depositing on the surface of CG network, the hydrogen bonds between COOH, OH, –NH_2_ of CG films and OH groups of HA crystals take the important role.” (p. 1215). Reprinted from Ref. [1067] with permission.

By means of the X-ray photo-electronic spectroscopy (XPS) technique, binding energies of Ca, P and O atoms were found to have some differences between nano-sized HA (Ca: 350.5 and 345.5; O: 530.2; P: 132.5 eV) and nano-sized HA/konjac glucomannan/chitosan biocomposite (Ca: 352.1 and 347.4; O: 531.2; P: 133.4 eV, respectively) [591]. Further measurements by FTIR and X-ray diffraction revealed that nano-sized HA was mainly linked with konjac glucomannan and chitosan by hydrogen bonding among OH^−^ and PO_4_^3−^ ions of HA and –C=O and –NH groups of konjac glucomannan and chitosan copolymer and there was a stable interface formed among the three phases in the biocomposite. Meanwhile, coordinate bonding might be formed between Ca^2+^ and –NH. Stable interfaces have been formed among the three phases in a biocomposite [591]. Hydrogen bonding between C=O groups of PDLLA and the surface P–OH groups of HA was discovered by XPS in another study [1065]. In HA/collagen biocomposites, a covalent bond formation between Ca^2+^ ions of HA and RCOO^−^ groups of collagen molecules was found by XPS [543]. Similar XPS observations were also made for several other CaPO_4_-based biocomposites and hybrid biomaterials [568,612,617]. All possible chemical interactions between HA crystals and bioorganic molecules in HA/chitosan-gelatin network films is shown in Figure 11 [1067].

The interaction and adhesion between CaPO_4_ fillers and respective matrixes have a significant effect on the properties of particulate filled reinforced materials, being essential to transfer the load among the phases and thus improve the mechanical performance of the biocomposites [283]. However, for a substantial amount of the aforementioned formulations, the interaction among the phases is mechanical in nature. This is because the matrix often consists of compounds with no functional groups or unsaturated bonds, which can form ionic complexes with the constituents of CaPO_4_. Obviously, less coupling exists between non-polar polymers and CaPO_4_ ceramic particles. Therefore, polymers with functional groups pendant to the polymer backbone, which can act as sites for bridging to CaPO_4_, are more promising in this respect [322].

In order to influence the interactions among the phases, various supplementary reagents are applied. Namely, if the primary effect of a processing additive is to increase the interaction between the phases, such additives can be regarded as coupling agents [1073]. These agents establish chemical bridges between the matrix and the fillers, promoting adhesion among the phases. In many cases, their effect is not unique; for example, it also might influence rheology of the composites [219]. In the case of CaPO_4_, a hexamethylene diisocyanate coupling agent was used to bind PEG/PBT (Polyactive™) block copolymers [228] and other polymers [1063] to HA filler particles. Thermogravimetric and infrared analysis demonstrated that the polymers were chemically bonded to the HA particles through the isocyanate groups, making it a suitable approach to improve the adhesion [1063]. Other researchers used glutaraldehyde as a cross-linked reagent [390,540,542,543,562,648,678,716,720,1074]. In addition, the interfacial bonding among CaPO_4_ and other components might be induced by silanes [204,205,228,333,576,1075,1076,1077,1078,1079,1080], zirconates [219,333,335,1081,1082,1083], titanates [219,333,1081,1084], phosphoric acid [579], alkaline pretreatment [799,801], PAA [1085] and some other chemicals. Furthermore, some polymers might be grafted onto the surface of CaPO_4_ particles [596]. Structural modifications of the polymeric matrices, for instance, with introduction of acrylic acid [186,204,205,228], have also proved to be effective methods. For example, application of polyacids as a bonding agent for HA/Polyactive™ composites caused the surface modified HA particles to maintain better contact with the polymer at fracture and improved mechanical properties [228]. The use of titanate and zirconate coupling agents appeared to be very dependent on the molding technique employed [219]. Silane-coupled HA powders were tested before applying them as fillers in biodegradable composites. This treatment allowed HA withstanding the attack of aqueous solutions without impairing overall bioactivity [1076,1077,1078,1079,1080]. Besides, a chemically modified reinforcement phase-matrix interface was found to improve the mechanical properties of the biocomposites. The examples include chemically coupled HA/PE [204,205], chemically formed HA/Ca poly(vinylphosphonate) [280] and PLA/HA fibers [178]. These biocomposites are able to consume a large amount of energy in the fracture.

The action of some coupling agents was found to combine two distinct mechanisms: (i) crosslinking of the polymeric matrix (valid for zirconate and titanate coupling agents) and (ii) improvement of the interfacial interactions among the major phases of the biocomposites. This interfacial adhesion improvement appeared to be much dependent on the chemical nature (pH and type of metallic centre) of the coupling agents [333]. Several works claimed that silanes did interact with HA [204,269,1076,1077,1078]. It was shown that a silicon-containing inter-phase existed between HA and PE, which promoted the chemical adhesion between the HA particles and the polymer. A silane-coupling agent also facilitated penetration of PE into cavities of individual HA particles, which resulted in enhanced mechanical interlocking at the matrix-reinforcement interface [204,205].

Thus, the optimization of biocomposite properties by coupling agents is currently an important area of the research. The control and development of molecular-level associations of polymers with CaPO_4_ is suggested to be significant for the resulting mechanical responses in biocomposites. It appears that a fundamental molecular understanding of the interfacial behavior in biocomposites is an area not sufficiently addressed in literature. Various experimental characterization techniques using electron microscopy, vibrational spectroscopy, X-ray diffraction, scanning probe microscopy and others are used routinely to characterize these materials besides mechanical property characterization. In addition, atomic scale models for simulating the phase interaction and predicting responses in the novel material systems, where nanostructures and nano-interfaces are included, are important to understand and predict the load deformation behavior [1047].

**Figure 12:**
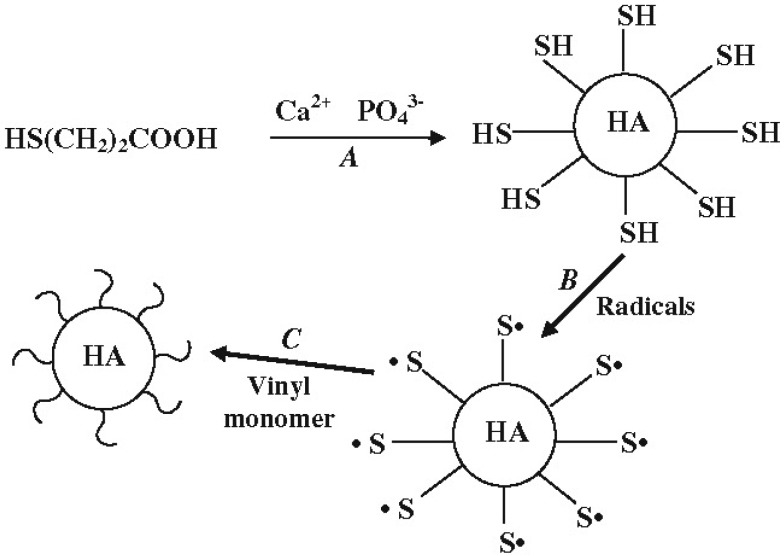
Surface modification of HA particles by grafting polymerization according to Lee *et al.*, [1092]: (**A**) Surface thiol functionalized HA; (**B**) Sulfur-centered radical on HA surface; (**C**) Surface grafting polymerization of ethylene glycol methacrylate phosphate. Reprinted from Ref. [63] with permission.

In addition to the aforementioned, the surface of CaPO_4_ might be modified as well [596,1081,1082,1083,1084,1085,1086,1087,1088,1089,1090,1091,1092,1093]. An interesting approach for HA surface modification was described [1092]. First, *in situ* synthesis of surface thiol-functionalized HA (HA-SH) was realized by adding 3-mercaptopropionic acid during hydrothermal synthesis of HA (Figure 12A). This was followed by grafting polymerization of ethylene glycol methacrylate phosphate by radical chain transfer generating the sulfur-centered radicals on the HA surfaces (Figure 12B), which initiated the surface grafting polymerization of ethylene glycol methacrylate phosphate (Figure 12C) [1092]. In certain cases, the surface functionalization of CaPO_4_ particles was found to decrease the bacterial adherence on their surface [1093]. Other examples might be found in literature [596,1081,1082,1083,1084,1085,1086,1087,1088,1089,1090,1091,1092,1093]. In general, the purpose of surface modifying is not only to guarantee the even distribution of CaPO_4_ particles at a high loading level in the matrix but also to prevent or delay the debonding process of CaPO_4_ particles from the matrix. Obviously, all surface modifiers must satisfy several biomedical requirements, such as no toxicity, good biocompatibility and no changes in the biological or physico-chemical properties of the fillers.

Addition of adhesion promoting agents might be an alternative to improve the interaction between the fillers and the matrix. For example, Morita *et al.* incorporated 4-methacryloyloxyethyl trimellitate anhydride to promote adhesion of the polymer to HA [1094]. In another study, a phosphoric ester was added to the liquid component of the formulation [1095]. Both the strength and the affinity index of biocomposites were found to increase, probably due to the effects of co-polymerization.

Possible interactions between BCP and HPMC have been investigated in IBS composites [838,839,1096]. After mixing, there was a decrease in the mean diameter of BCP granules and this influenced the viscosity of the paste. Dissolution of grain boundaries of β-TCP crystals and precipitation of CDHA on HA crystal surface were found during the interaction. Both phenomena were responsible for the observed granulometric changes [838,839]; however, within the sensitivity of the employed measurement techniques, no chemical bonding between BCP and HPMC was detected [1096].

A co-precipitation technique was used to prepare CDHA/chitosan biocomposites [746]. Growth of CDHA crystals was inhibited by organic acids with more than two carboxyl groups, which strongly bind to CDHA surfaces via COO–Ca bonds. Transmission electron microscopy images revealed that CDHA formed elliptic aggregates with chemical interactions (probably coordination bond) between Ca on its surface and amino groups of chitosan; the nano-sized crystals of CDHA were found to align along the chitosan molecules, with the amino groups working as the nucleation sites [746]. Formation of calcium cross-linked polymer carboxylate salts was suggested during setting of self-hardening (TTCP + DCPA)/polyphosphazane biocomposites; a chemical involvement of the polymer in the setting process was concluded based on the results of pH monitoring [490,491,492].

A chemical bond between the phases was presumed in PCL/HA composites, prepared by the grafting technique [345]; unfortunately, no strong experimental evidences were provided. In another study, CDHA/poly(α-hydroxyester) composites were prepared by a low temperature chemical route [320]. In that study, pre-composite structures were prepared by combining α-TCP with PLA, PLGA and copolymers thereof. The final biocomposite was achieved by *in situ* hydrolysis of α-TCP to CDHA performed at 56 °C either in solvent cast or pressed pre-composites. That transformation occurred without any chemical reaction between the polymer and CaPO_4_, as it was determined by FTIR spectroscopy [320].

In nearly every study on HA/carbon nanotubes biocomposites, the nanotubes were functionalized before combining them with HA. Most researchers did this by oxidation [239,240,241,980,981], although non-covalent functionalizing with sodium dodecylsulfate [980] and coating the nanotubes by a polymer [1097] before combining them with HA were also reported. Several studies by transmission electron microscopy revealed evidences that the functionalization enhanced interaction between carbon nanotubes and HA [980,981,1098].

For CaPO_4_-based biocomposites able to sustain a high-temperature sintering (valid for the formulations consisting of inorganic components only), an inter-diffusion of chemical elements might take place among the phases. Such effect was detected by energy-dispersive X-ray spectroscopy in HA/TiO_2_ biocomposite particles with partial formation of calcium titanates; this process was found to be favorable to enhancing the cohesive strength of particles in the composite coating [1099]. Similar high-temperature interactions between HA and zirconia [849,872], as well as between HA and Ti [603,927,928,930,939,942] were also detected. Namely, lower Ti content composites sintered at 1200 °C showed main crystalline phases as CaTiO_3_, CaO and Ti*_x_*P*_y_*, while an increase in Ti content to 50 vol.% revealed Ti_2_O and residual α-Ti as additional phases. Thus, the chemical interactions between HA and Ti were expressed by the following unbalanced illustrative scheme [928]:

Ti + Ca_10_(PO_4_)_6_(OH)_2_ → CaTiO_3_ + CaO + Ti_x_P_y_ + (Ti_2_O) + (Ca_4_P_2_O_9_) + H_2_O (1)

Such undesired interactions between Ti and HA could be minimized if Ti particles were coated by silica [943].

Besides, partial decomposition of HA and formation of various calcium aluminates were detected in HA/Al_2_O_3_ biocomposites after sintering at 1200–1300 °C. This has been attributed to the diffusion of Ca^2+^ from HA into the alumina matrix and the depletion of Ca^2+^ from HA leads to the decomposition of HA into β-TCP. All these processes influence the mechanical strength of the biocomposites [883,884,885,886,887,1099].

## 6. Bioactivity and Biodegradation

The continuous degradation of an implant causes a gradual load transfer to the healing tissue, preventing stress-shielding atrophy and stimulates the healing and remodeling of bones. Some requirements must be fulfilled by the ideal prosthetic biodegradable materials, such as biocompatibility, adequate initial strength and stiffness, retention of mechanical properties throughout sufficient time to assure its biofunctionality and non-toxicity of the degradation by-products [125]. In most cases, bioactivity (*i.e.*, ability of bonding to bones) of biologically relevant CaPO_4_ reinforced by other materials is lower than that of pure CaPO_4_ [148,1100].

In general, both bioactivity and biodegradability of any biocomposite and/or hybrid biomaterial are determined by the same properties of the constituents. Both processes are very multi-factorial because, during implantation, the surface of any graft contacts with biological fluids and, shortly afterwards, is colonized by cells. Much more biology, than chemistry and material science altogether, is involved into these very complex processes and many specific details still remain unknown. In addition, biodegradation of all components of biocomposites occurs simultaneously and the obtained byproducts might influence both the entire process and biodegradation of each component. For example, in the case of biocomposites prepared from polyesters and TCP, hydrolysis reactions of the ester bonds, acid dissociation of the carboxylic end groups, dissolution of TCP and buffering reactions by the dissolved phosphate ions occur simultaneously [1101,1102,1103,1104]. In such cases, basic TCP buffer the acidic degradation products of polyesters, thus reducing autocatalysis and delaying polymer degradation. This is why both pH and mass drops occurred at earlier degradation time points for the pure polymer samples than for the corresponding composites. However, this is not always the case. Namely, studies are available, in which the presence of CaPO_4_ did not have an effect on the degradation rate of the polymer matrix [1105,1106,1107]. Therefore, to simplify the task, biodegradation of the individual components should be considered independently. Namely, an *in vitro* biodegradation of the biologically relevant CaPO_4_ might be described by their chemical dissolution in slightly acidic media (they are almost insoluble in alkaline solutions [74,75]), which, in the case of CDHA, might be described as a sequence of four successive chemical Equations (2)–(5) [1108,1109]:

Ca_10−*x*_(HPO_4_)*_x_*(PO_4_)_6−*x*_(OH)_2−*x*_ + (2–*x*)H^+^ = Ca_10−*x*_(HPO_4_)*_x_*(PO_4_)_6−*x*_(H_2_O)_2−*x*_^(2−*x*)+^ (2)

Ca_10−*x*_(HPO_4_)*_x_*(PO_4_)_6−*x*_(H_2_O)_2−*x*_^(2^_−_*^x^*^)+^ = 3Ca_3_(PO_4_)_2_ + (1–*x*)Ca^2+^ + (2–*x*)H_2_O (3)

Ca_3_(PO_4_)_2_ + 2H^+^ = Ca^2+^ + 2CaHPO_4_ (4)

CaHPO_4_ + H^+^ = Ca^2+^ + H_2_PO_4_^−^ (5)

Biodegradability of polymers generally depends on the following factors: 1) chemical stability of the polymer backbone, 2) hydrophobicity of the monomer, 3) morphology of the polymer, 4) initial molecular weight, 5) fabrication processes, 6) geometry of the implant, 7) properties of the scaffold such as porosity and pore diameter [262]. A summary on degradation of PLA and PGA, as well as that of SEVA-C is available in literature Ref. [125] (p. 798 and p. 803, respectively), where the interested readers are referred to.

Concerning *in vivo* studies, biodegradation of HA/PLLA and CDHA/PLLA biocomposite rods in subcutis and medullary cavities of rabbits were investigated mechanically and histologically; the degradation was found to be faster for the case of using uncalcinated CDHA instead of calcinated HA [1110]. In a more detailed study, new bone formation was detected at 2 weeks after implantation, especially for formulations with a high HA content [1111]. More to the point, a direct contact between bones and these composites without intervening fibrous tissue was detected in this case [1111,1112]. Both SEVA-C and SEVA-C/HA biocomposites were found to exhibit a non-cytotoxic behavior [1113,1114], inducing a satisfactory tissue response when implanted as shown by *in vivo* studies [1114]. Furthermore, SEVA-C/HA biocomposites induced a positive response on osteoblast-like cells to what concerned cell adhesion and proliferation [1113]. An *in vivo* study on biodegradation of microspheres (PLGA, gelatin and PTMC were used)/CaPO_4_ biocomposites revealed that they exhibited microsphere degradation after 12 weeks of subcutaneous implantation, which was accompanied by compression strength decreasing [1115]. Interestingly that the amount of CaPO_4_ in biocomposites was found to have a greater effect on the early stages of osteoblast behavior (cell attachment and proliferation) rather than the immediate and late stages (proliferation and differentiation) [1116].

Both *in vitro* (the samples were immersed into 1% trypsin/phosphate-buffered saline solution at 37 °C) and *in vivo* (implantation of samples into the posterolateral lumbar spine of rabbits) biodegradation were investigated for nano-sized HA/collagen/PLA biocomposites [551]. The results demonstrated that weight loss increased continuously *in vitro* with a reduction in mass of ~20% after 4 weeks. During the experimental period *in vitro*, a relative rate of reduction of the three components in this material was shown to differ greatly: collagen decreased the fastest, from 40% by weight to ~20% in the composite; HA content increased from 45% to ~60%; while the amount of PLA changed a little. *In vivo*, the collagen/HA ratio appeared to be slightly higher near the transverse process than in the central part of the intertransverse process [551]. Hasegawa *et al.*, [1117] performed *in vivo* study, spanning over a period of 5–7 years, on high strength HA/PLLA biocomposite rods for the internal fixation of bone fractures. In that work, both uncalcined CDHA and calcined HA were used as reinforcing phases in PLLA matrix. Those composites were implanted in the femur of 25 rabbits. It was found that the implanted materials were resorbed after 6 years of implantation. The presence of remodeled bone and trabecular bone bonding was the significant outcome. These data clearly demonstrate a biodegradation independence of various components of biocomposites.

## 7. Some Challenges and Critical Issues

The scientific information summarized in this review represents the developments of CaPO_4_-based biocomposites and hybrid biomaterials from a variety of approaches, starting from conventional ones to tissue engineering. Such formulations combined with osteoconductive, osteoinductive factors, and/or osteogenic cells have already gained much interest as a new and versatile class of biomaterials and are perceived to be beneficial in many aspects as bone grafts [22,1118]. However, current applications of these biomaterials in medicine and surgery are still remarkably less than might be expected. In many biomedical applications, research and testing of such formulations have been introduced and highly developed but only in a very few cases an industrial production and commercial distribution of medical devices partially or entirely made of biocomposites have started. The medical application of biocomposites and hybrid biomaterials requires a better understanding of the objectives and limitations involved. Recently, the main critical issues have been summarized as follows [197]:

- There are not enough reliable experimental and clinical data supporting the long-term performance of biocomposites with respect to monolithic traditional materials;
- The design of biocomposites and hybrid biomaterials is far more complex than that of conventional monolithic materials because of the large number of additional design variables to be considered;
- The available fabrication methods may limit the possible reinforcement configurations, may be time consuming, expensive, highly skilled and may require special cleaning and sterilization processes;
- There are no satisfactory standards yet for biocompatibility testing of the biocomposite implants because the ways in which the components of any biocomposite interact to living tissues are not completely understood;
- There are no adequate standards for the assessment of biocomposite fatigue performance because the fatigue behavior of such materials is far more complex and difficult to predict than that of traditional materials [197].

In addition, much work needs to be done in the analysis of cells and their different behaviors with regard to their interactions with CaPO_4_/polymer biocomposites. Important but unresolved questions are: (1) What is/are the mechanism(s) by which such biocomposites promote cell proliferation and differentiation? (2) How can we determine the pathways? Future studies will focus on the ability to functionalize the surfaces of CaPO_4_/polymer biocomposites with molecules of different natures and dimensions by means of their attachment to cells that will enable them to act selectively on biological molecules such as proteins and peptides [264].

On the other hand, in spite of an enormous progress in biocomposite processing, to achieve the desired characteristics researchers still need to develop more advanced technologies to fabricate a bone-resembling hierarchical organization over several length scales. Development of novel grafting materials depends on the progress in research into the structure of natural bones. The key issues are not only to understand the fundamentals of biomineralization but also to translate such knowledge into practical synthetic pathways to produce better bone grafts. Unfortunately, when it comes to the fabrication of biocomposites, mimicing natural bones from the nanometer to the micrometer dimensions, there are many key issues, including control of morphology, incorporation of foreign ions, interaction with biomolecules and assembly of the organic and inorganic phases, which are still not well understood. A processing gap between the lower-level building units and the higher-order architecture could severely limit the practical application of current CaPO_4_-based biocomposites and hybrid biomaterials. Therefore, further substantial research efforts have been outlined to address the following key challenges [22]:

- Optimizing biocomposite processing conditions;
- Optimization of interfacial bonding and strength equivalent to natural bone;
- Optimization of the surface properties and pore size to maximize bone growth;
- Maintaining the adequate volume of the construct *in vivo* to allow bone formation to take place;
- Withstanding the load-bearing conditions;
- Matching the bioresorbability of the grafts and their biomechanical properties while forming new bone;
- Understanding the molecular mechanisms by which the cells and the biocomposite matrix interact with each other *in vivo* to promote bone regeneration;
- Supporting angiogenesis and vascularization for the growth of healthy bone cells and subsequent tissue formation and remodeling.

The aforementioned critical issues have to be solved before a widespread commercial use of CaPO_4_-based biocomposites and hybrid biomaterials can be made in surgery and medicine.

## 8. Conclusions

All types of calcified tissues of mammals appear to possess complex hierarchical structures and, from the material point of view, they are multi-phase organic/inorganic biocomposites. Their mechanical properties are outstanding (considering weak constituents from which they are assembled) and far beyond those, that can be achieved using the same synthetic materials with present technologies. This is because biological organisms produce biocomposites that are organized in terms of both composition and structure, containing both brittle CaPO_4_ and ductile bioorganic components in very complex structures, hierarchically organized at the nano-, micro- and meso-levels. Additionally, the calcified tissues are always multifunctional: for example, bones provide structural support for the body and are responsible for blood cell formation. The third defining characteristic of biological systems, in contrast with current synthetic systems, is their self-healing ability, which is nearly universal in nature. These complex structures, which have risen from millions of years of evolution, inspire materials scientists in the design of novel biomaterials [1119].

Obviously, no single-phase biomaterial is able to provide all the essential features of bones and/or other calcified tissues and therefore, there is a great need to engineer multi-phase biocomposites with the structure and composition mimicking those of natural bones. This resulted in an idea of adding CaPO_4_ to polymers, which further led from the bioinert monolithic materials used in the 1970s and 1980s to the bioactive biodegradable biocomposites used now as modulus-matched biomaterials and since recently as the scaffolds for tissue engineering. This approach resulted in applications of conventional composite manufacturing techniques to the field of biomaterials and the studies summarized in this review have shown that the proper combination of a ductile matrix with a brittle, hard and bioactive CaPO_4_ filler offers many advantages for biomedical applications. Namely, the desirable properties of some components can compensate for a poor mechanical behavior of CaPO_4_ bioceramics, while in turn the desirable bioactive properties of CaPO_4_ improve those of other phases, thus expanding the possible application of each material within the body [66,67]. However, the reviewed literature clearly indicates that among possible types of CaPO_4_-based biocomposites and hybrid biomaterials only simple, complex and graded ones, as well as fibrous, laminar and particulate ones (see Section 2. “General Information and Knowledge” for details) have been investigated. Presumably, a future progress in this subject will require concentrating efforts on elaboration and development of both hierarchical and hybrid biocomposites. Furthermore, following the modern tendency of tissue engineering, a novel generation of CaPO_4_-based biocomposites and hybrid biomaterials should also contain a biological living part.

To finalize, the future of the CaPO_4_-based biocomposites and hybrid biomaterials is now directly dependent on the formation of multidisciplinary teams composed of experts but primarily experts ready to collaborate in close collaboration with others and thus be able to deal efficiently with the complexity of the human organism. The physical chemistries of solids, solid surfaces, polymer dispersion and solutions, as well as material-cell interactions are among the phenomena to be tackled. Furthermore, much work remains to be done on a long way from a laboratory to clinics and the success depends on the effective co-operation of clinicians, chemists, biologists, bioengineers and materials scientists.

## Abbreviations

A-W: apatite-wollastonite
BMP: bone morphogenetic protein
BSA: bovine serum albumin
CMC: carboxymethylcellulose
EVOH: a copolymer of ethylene and vinyl alcohol
IBS: injectable bone substitute
HDPE: high-density polyethylene
HPMC: hydroxypropylmethylcellulose
PA: polyamide
PAA: polyacrylic acid
PBT: polybutyleneterephthalate
PCL: poly(ε-caprolactone)
PDLLA: poly(D,L-lactic acid)
PE: polyethylene
PEEK: polyetheretherketone
PEG: polyethylene glycol
PGA: polyglycolic acid
PHB: polyhydroxybutyrate
PHBHV: poly(hydroxybutyrate*-co-*hydroxyvalerate)
PHEMA: polyhydroxyethyl methacrylate
PLA: polylactic acid
PLGA: poly(lactic*-co-*glycolic) acid
PLGC: *co*-polyester lactide*-co-*glycolide*-co-*ε-caprolactone
PLLA: poly(L-lactic acid)
PMMA: polymethylmethacrylate
PP: polypropylene
PPF: poly(propylene*-co-*fumarate)
PSZ: partially stabilized zirconia
PTMC: poly(trimethylene carbonate)
PU: polyurethane
PVA: polyvinyl alcohol
PVAP: polyvinyl alcohol phosphate
SEVA-C: a blend of EVOH with starch
